# Emerging Energy Harvesters in Flexible Bioelectronics: From Wearable Devices to Biomedical Innovations

**DOI:** 10.1002/smsc.202300148

**Published:** 2024-01-29

**Authors:** Swarup Biswas, Sang Won Lee, Yongju Lee, Hyo-Jeong Choi, Jianjun Chen, Xiao Yang, Yuxuan Du, Natashya Falcone, Natan Roberto de Barros, Sung-Min Lee, Hyeok Kim, Ali Khademhosseini, Yangzhi Zhu

**Affiliations:** ^1^ School of Electrical and Computer Engineering Center for Smart Sensor System of Seoul (CS4) University of Seoul Seoul 02504 Republic of Korea; ^2^ Terasaki Institute for Biomedical Innovation Los Angeles CA 90064 USA; ^3^ Department of Chemical and Environmental Engineering University of California, Riverside Riverside CA 92521 USA; ^4^ Department of Mechanical Engineering City University of Hong Kong Hong Kong 999077 China; ^5^ Mork Family Department of Chemical Engineering & Materials Science Viterbi School of Engineering University of Southern California Los Angeles CA 90007 USA; ^6^ Department of Electrical Engineering Hanyang University Seoul 04763 Republic of Korea

**Keywords:** biomedical devices, energy harvesters, flexible bioelectronics, personalized healthcare wearable electronics

## Abstract

Flexible bioelectronic devices have attracted immense interest in the biomedical field because of their wearability, biocompatibility, diverse functionalities, and ease of personalization. Recently, flexible energy harvesters have been developed, and attempts have been made to integrate them with such devices, enabling them to operate without external batteries or power supply. In this review, the latest advances in flexible energy harvesters and their use in wearable devices are discussed. Energy harvesters for versatile biomedical applications are summarized. Finally, the challenges and future perspectives of self‐powered biomedical systems that enable wearable healthcare monitoring and management are outlined.

## Graphical abstract

Flexible energy harvesters have been developed for integrating with various wearable medical devices to enable power‐free operation. This review discusses the recent advances in flexible energy harvesters, including versatile energy harvesters as well as their biomedical applications. Challenges and future perspectives of self‐powered biomedical systems are outlined.

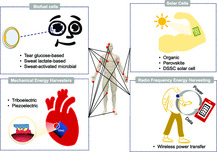

## 1 Introduction

The field of flexible electronics has witnessed accelerated progress in the last decade.^[^ ^1^, ^2^ ^]^ Flexible electronics have been designed and developed to replace the conventional rigid and bulky electronics that seamlessly interface with the human body for versatile biomedical innovations, including wearable sensors,^[^ ^3^, ^4^, ^5^, ^6^ ^]^ stretchable displays,^[^ ^7^, ^8^ ^]^ and digital medicine.^[^ ^9^, ^10^ ^]^ With the rapid development of machine learning, additive manufacturing, and semiconductor production, flexible electronics have evolved into a more miniaturized, personalized, and accurate toolbox used in intelligent healthcare management, smart agriculture, and the Internet of Medical Things.

Albeit their long‐lasting acceleration in the current stage, developing a sustainable and reliable power supply for flexible electronics in human‐centered healthcare is still a challenge. Replaceable batteries are currently the most widely used option to power such devices. However, their natural rigidity fails to adapt to the conformity of dynamic and nonplanar skin surfaces, limiting their utilization in on‐body applications. Furthermore, most batteries are not disposable, so they may become electronic waste at the end of their shelf‐life period and pollute the environment or even harm the human body.

Emergingly, extensive research has been dedicated to flexible energy harvesters for environmentally friendly and sustainable energy supplies.^[^ ^11^, ^12^ ^]^ Such energy harvesting systems have been explored to harness different forms of energy, including mechanical, chemical, thermal, and solar power, for flexible electronic devices. Therefore, based on these specific energy sources, flexible energy harvesting systems have been summarized into four major types: biofuel cells (BFCs), mechanical energy harvesters, radio frequency (RF) energy harvesters, and solar cells. BFCs utilize enzymes or microorganisms as catalysts to transform chemical energy into electrical energy, enabling them a promising method for harvesting chemical energy stored in the human body.^[^ ^13^ ^]^ Thermoelectric generators can efficiently convert heat inside the human body into electric power using the temperature difference between the human body and the surrounding environment,^[^ ^14^ ^]^ while piezoelectric and triboelectric generators have been well‐suited for harnessing mechanical energy during human motions.^[^ ^15^, ^16^ ^]^ However, the development and application of these technologies pose several difficulties. It necessitates the seamless integration of cutting‐edge design concepts, microfabrication methods, and material science. When addressing the intricate needs of wearable and biomedical applications, energy economy, device adaptability, and biocompatibility concerns all take on a crucial role.

Harvesting multiple forms of energy within a single system can further improve energy harvesting efficiency in real‐life scenarios. Such self‐powered strategies are the emerging trend in next‐generation flexible electronics. In order to explore the dynamic landscape of emerging energy harvesters in flexible bioelectronics, encompassing a wide range of devices and their potential applications and to highlight recent advancements and breakthroughs in this field, a thorough summary of current energy harvesting systems and their applications in biomedical innovations is necessary. This summary can be very instructive for researchers in wearable biomedical systems.

**Figure 1:**
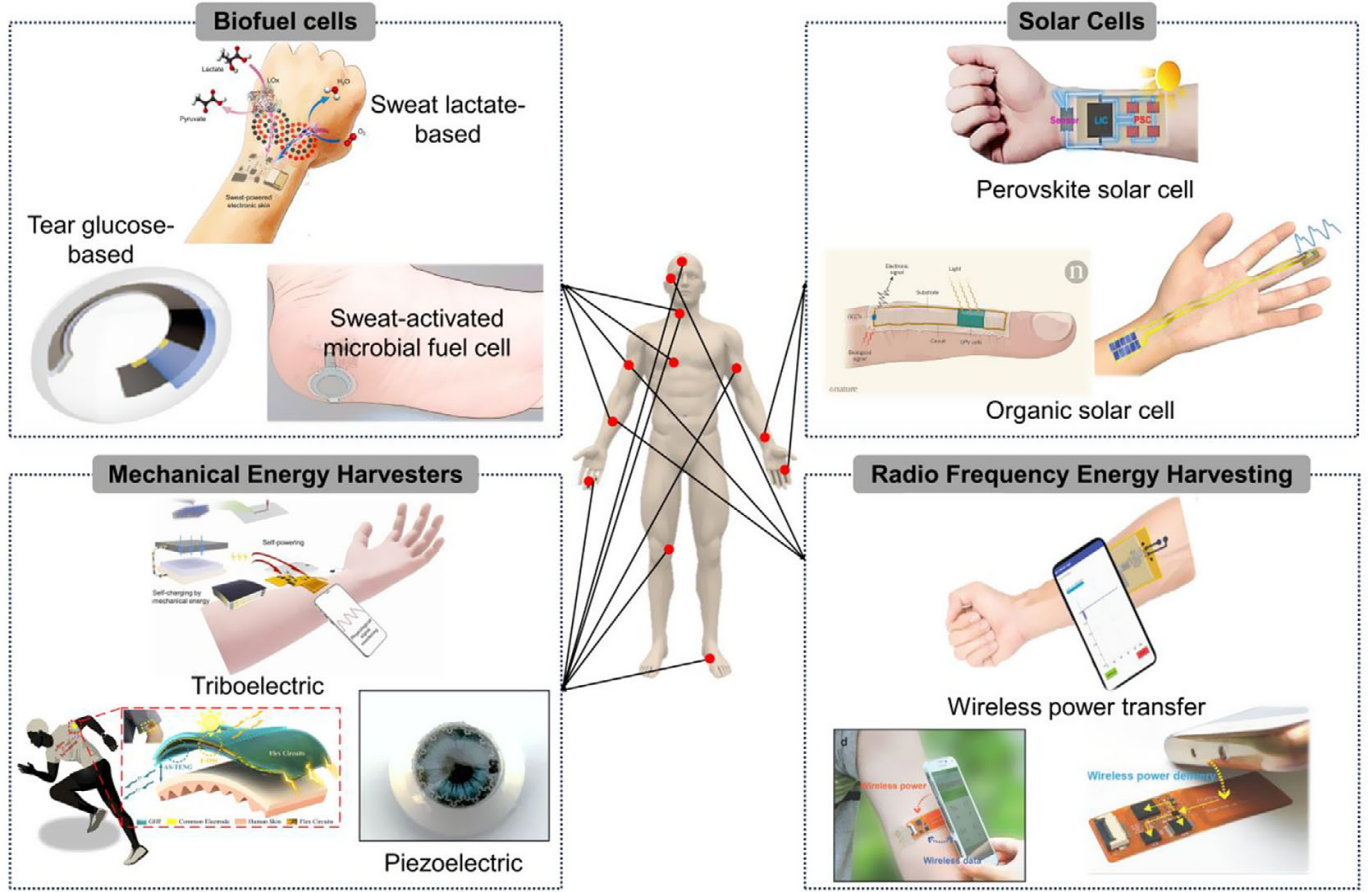
Recent advances in flexible energy harvesters for biomedical innovations. This work summarizes four representative energy harvesters: BFCs, solar cells, mechanical energy harvesters, and RF energy harvesters. Images for “Biofuel Cells”: Top: Reproduced with permission.^[^ ^43^ ^]^ Copyright 2020, American Association for the Advancement of Science. Bottom left: Reproduced with permission.^[^ ^180^ ^]^ Copyright 2022, American Chemical Society. Bottom right: Reproduced with permission.^[^ ^181^ ^]^ Copyright 2022, Elsevier. Images for “Solar cells”: Top: Reproduced with permission.^[^ ^149^ ^]^ Copyright 2019, Elsevier. Bottom left: Reproduced with permission.^[^ ^138^ ^]^ Copyright 2018, The Authors, published by Springer Nature. Bottom right: Reproduced under the terms of the CC‐BY Creative Commons Attribution 4.0 International license (https://creativecommons.org/licenses/by/4.0).^[^ ^183^ ^]^ Copyright 2021, Springer Nature. Images for “Mechanical energy harvesters”: Top: Reproduced with permission.^[^ ^90^ ^]^ Copyright, 2022, Elsevier. Bottom left: Reproduced with permission.^[^ ^185^ ^]^ Copyright 2020, Elsevier. Bottom right: Reproduced under the terms of the CC‐BY Creative Commons Attribution 4.0 International license (https://creativecommons.org/licenses/by/4.0).^[^ ^184^ ^]^ Copyright 2023, The Authors, published by Springer Nature. Images for “RF energy harvesters”: Top: Reproduced with permission.^[^ ^131^ ^]^ Copyright 2023, Wiley‐VCH. Bottom left and right: Reproduced with permission.^[^ ^165^ ^]^ Copyright 2019, Wiley‐VCH.

In this review, we first provide an in‐depth overview of different flexible energy harvesters based on various principles or mechanisms (**Figure** **1**). Next, representative applications of flexible energy harvesters are described. We also discuss the current challenges and future outlook of this field.

## 2 Energy Harvesters on Flexible Substrate

Energy harvesters on flexible substrates are at the bleeding edge of materials science, electronics, and renewable energy technology. This subtopic delves into the interesting world of energy harvesting systems embedded in flexible materials, suggesting a paradigm change in how we power and sustain electronic gadgets. These flexible substrates offer the possibility of powering autonomous and wearable devices without the use of standard batteries by converting ambient energy sources such as biological, motion, light, and heat into electrical power. This advancement not only increases device longevity but also minimizes environmental impact by reducing battery waste.

### 2.1 Biofuel Cells

BFCs are a promising candidate for wearable power sources as their bioenergy harvesting through redox reactions is highly efficient.^[^ ^13^, ^17^, ^18^ ^]^ BFCs are usually composed of cathodes, anodes, and electrolytes. Biofuels, such as lactate, glucose, or ethanol in the electrolytes, can serve as fuel sources.^[^ ^19^, ^20^, ^21^ ^]^ Enzymes, microbes, or organelles immobilized at the electrodes function as biocatalysts.^[^ ^22^, ^23^, ^24^ ^]^ The biocatalyst promotes biochemical energy conversion in biofluids under specific physiological conditions.^[^ ^25^, ^26^ ^]^ In summary, the operating mechanism of BFCs involves the oxidation of biofuel with the assistance of a biocatalyst, resulting in the generation of electrons. These generated electrons can flow to the cathode via an external circuit, facilitating the reduction of oxidants (e.g., O_2_) to develop a net electrical current.^[^ ^27^ ^]^ Thus, the power output performance of BFCs primarily relies on biofuel sources in bodily fluids and the electrochemical interaction between the biocatalysts and the electrode surfaces.^[^ ^28^ ^]^

For highly efficient energy conversion, it is crucial to establish efficient electron transport between the biocatalysts and electrodes. The biocatalysts’ wiring and redox mediator approaches are employed to address the challenge that most enzyme‐based biocatalysts cannot directly shuttle electrons using the supporting electrodes.^[^ ^29^, ^30^, ^31^, ^32^ ^]^ Redox mediators are utilized as electron carriers between the biocatalyst and the electrode surface.^[^ ^33^ ^]^ However, this strategy has certain limitations owing to the potential toxicity concerns associated with specific redox mediators, which can lead to leakage issues. Conductive carbon nanomaterials, such as carbon nanotubes (CNTs), carbon nanodots, and graphene, are highly promising connecting materials for incorporating biocatalysts.^[^ ^34^, ^35^, ^36^, ^37^ ^]^ Apart from the challenges of electrochemical communication, a constant power supply is essential, which can be realized using sustainable and continuously available biofuel sources such as blood, sweat, interstitial fluid (ISF), saliva, and tears. Furthermore, BFCs require direct and close contact with the human body to ensure the availability of biofuel sources, even during dynamic deformations. These requirements and the emphasis on wearing comfort underscore the significance of mechanical resilience, stretchability, and flexibility in BFCs.^[^ ^38^ ^]^

**Figure 2:**
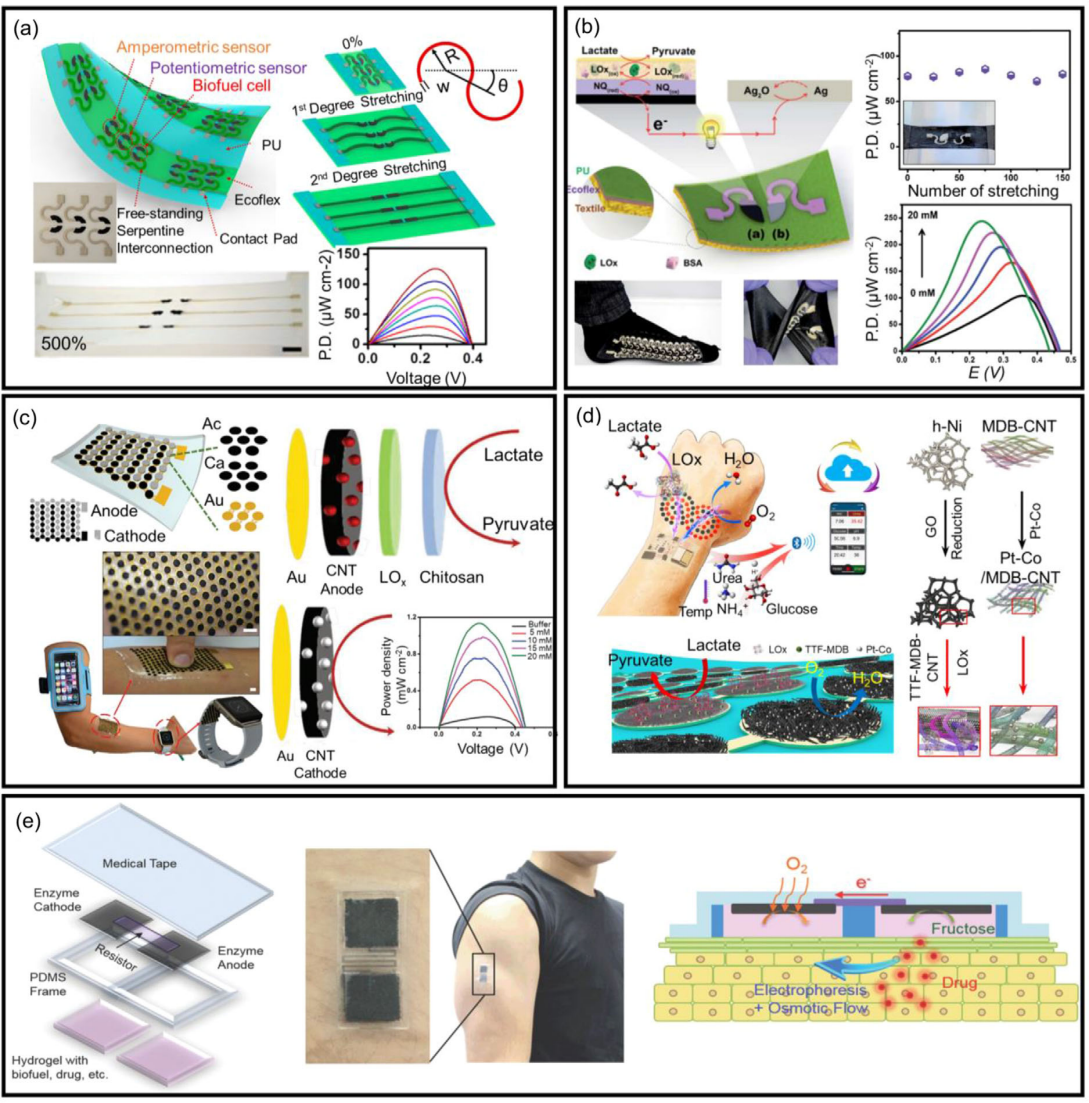
BFCs in wearable biomedical devices. a) Large‐scale‐printed stretchable BFC device having a serpentine structure. Reproduced with permission.^[^ ^40^ ^]^ Copyright 2015, American Chemical Society. b) Textile‐based stretchable BFCs. Reproduced with permission.^[^ ^41^ ^]^ Copyright 2016, Royal Society of Chemistry. c) Stretchable electronic skin‐type island–bridge BFCs. Reproduced with permission.^[^ ^42^ ^]^ Copyright 2017, Royal Society of Chemistry. d) BFCs with a unique integration from 0D to 3D nanomaterials on bioanodes and cathodes. Reproduced with permission.^[^ ^43^ ^]^ Copyright 2020, American Association for the Advancement of Science. e) Built‐in BFCs for iontophoresis‐assisted drug delivery. Reproduced with permission.^[^ ^51^ ^]^ Copyright 2015, Wiley‐VCH.

Wearable BFCs based on temporary transfer tattoos can convert biochemical energy from sweat lactate to electrical energy with a power density of up to 70 μW cm^−2^.^[^ ^39^ ^]^ Since dynamic skin deformations occur during practical applications, the conductive support's cracking and the biocatalyst's exfoliation within BFCs will deteriorate their performance. To address this concern, functional stress‐enduring material and rational configuration design were used to realize flexible and stretchable BFCs. Screen‐printable inks were used to fabricate a stretchable BFC (**Figure** **2**a).^[^ ^40^ ^]^ The exceptional mechanical and electrical properties of CNTs and the elastomeric capabilities of the polyurethane binder imparted considerable stretchability to the BFCs. The combination of configuration pattern and material design enabled the resultant BFCs to tolerate a variety of mechanical deformations, such as linear stretching, torsional twisting, and indentation stress. This BFC could withstand up to 500% stresses while maintaining structural integrity and electrochemical performance. Furthermore, the power density showed an increase in the levels of glucose. The combination of advanced ink formulations and serpentine structure design was further used to develop textile‐based BFCs (Figure 2b).^[^ ^41^ ^]^ The synergistic effects endowed high stretchability for textile‐based bioelectronic devices, presenting a stable power output after 100 stretching iterations under 100% strain. Textile‐based BFCs were also used on soft commercial items such as socks, undergarments, and textile straps. In the presence of 20 mm lactate, the BFC exhibited a 0.46 V of open‐circuit voltage and a 250 μW cm^−2^ of maximum power density.

Integrating BFCs into wearable bioelectronic devices requires a power density on the order of mW to satisfy the power consumption of commercial radio transmitters. An island–bridge architecture was designed to improve power density. The conductive serpentine structure functioned as a bridge to electrically connect the islands of densely packed electrodes, resulting in the ability to withstand mechanical deformation and prevent short circuits (Figure 2c).^[^ ^42^ ^]^ The anode islands were composed of compact 3D CNT‐naphthoquinone, and the cathodes consisted of densely packed 3D CNT‐silver oxide. Such functionalization boosted the energy‐harvesting performance from human sweat with a power density (≈1.2 mW cm^−2^) at 0.2 V. The stretchable BFC generated ≈1 mW during exercise, sufficient for powering simple electronic components. Yu et al. reported a biofuel‐based energy harvester for on‐body applications, which performed multiplexed continuous biosensing and wireless data transmission to a smartphone app via Bluetooth. (Figure 2d).^[^ ^43^ ^]^ To increase the energy density of BFCs, they fabricated a monolithic integration of 0D–3D conductive nanomaterials. For a bioanode, a lactate oxidase was immobilized at the host, consisting of modified hierarchical nickel, reduced graphene oxide films, and a modified CNT (MDB‐TTF‐CNT) electrode array. The biocathode was composed of modified CNT with Pt‐based nanoparticles. This unique integration imparted 3.5 mW cm^−2^ of power density to the BFCs through human body fluids. The high‐energy‐harvesting capabilities of BFCs showed promising potential in wearable applications such as self‐powered biosensors and controlled medication administration.^[^ ^44^, ^45^ ^]^ The high power output of the BFCs enabled real‐time and wireless data transmission with chemical information (e.g., urea, NH_4_^+^, glucose, and pH) and physical information (e.g., temperature, hydration, and pressure).^[^ ^43^ ^]^ For example, Bandodkar et al. devised microfluidic‐based BFCs that can support multiplexed monitoring of lactate, glucose, chloride concentration, pH, sweat rate, and total sweat loss.^[^ ^46^ ^]^ The BFCs produced an electrical current proportional to lactate and glucose concentrations, and such a current response was converted to a voltage‐based electrical signal by connecting a resistor across the sensor. The pH measurements in the colorimetric platform could compensate for the pH effect to capture accurate data under different physiological conditions. Furthermore, such viable BFCs could be utilized to power near‐field communication (NFC) for wireless health information transmission, which gleans critical insights into overall health.^[^ ^47^, ^48^ ^]^

Overall, BFCs can potentially be used for controlled drug delivery by loading drugs onto the electrolyte or electrodes.^[^ ^49^, ^50^ ^]^ A stretchable organic patch with built‐in BFCs could achieve current‐assisted ascorbyl glucoside and rhodamine B penetration (Figure 2e).^[^ ^51^ ^]^ The fructose/O_2_ BFCs generated a transdermal ionic current, and the corresponding osmotic flow promoted drug delivery by electrophoretic movement. According to emergent studies, BFCs indicated a promising future of self‐powered flexible and wearable electronic devices having multiple functionalities.

### 2.2 Mechanical Energy Harvesters

The increased demand for portable, wearable, and flexible electronic devices for long‐term power generation has fuelled research into alternate energy sources. Mechanical energy harvesters, which transform ambient mechanical energy, such as motion and vibrations, into electrical energy, have emerged as a good alternative. Mechanical energy harvesters provide sustainable and efficient energy to power electrical devices. Devices with mechanical energy harvesters are lightweight, small, and can gather energy from various sources, including human motions and vibrations. Such harvesters can generate power from walking or joint movements, enabling them to be used for self‐powered flexible sensors that monitor vital signs, track physical activity, or aid rehabilitation. These self‐powered devices have the benefit of being able to operate continuously without the requirement for regular battery changes or recharging.

Furthermore, mechanical energy harvesters generate electricity from physiological or organ movements, offering a long‐term energy source for bioelectronic implants. This allows devices, such as pacemakers, neurostimulators, and medication delivery systems, to work autonomously within the body, decreasing the need for invasive treatments and enhancing patient comfort. Mechanical energy harvesters have also been used for the applications of environmental monitoring. Sensor integration into wearable or deployable devices enables the collection of vital data such as vibrations, temperature, or airflow. These sensors may be used to detect environmental threats early and even track the health of plants and animals in ecological research.

One of the most exciting aspects of power generator technology is that various structural properties might be utilized for flexible electronics. These devices are primarily based on two modes: piezoelectricity and triboelectricity. Therefore, this section focuses on recent advances in energy‐harvesting techniques, such as piezoelectric nanogenerators (PENGs) and triboelectric nanogenerators (TENGs), for flexible electronics.

#### 2.2.1 Piezoelectric Nanogenerators

Piezoelectric materials have the singular capacity to generate an electric charge under mechanical stress conditions. PENGs derived based on this ability can transform mechanical stimuli into electrical energy, such as bending, twisting, and stretching. Its operation is based on the piezoelectric effect of specific materials, in which mechanical stress causes an electric charge imbalance inside the material. The selection of appropriate piezoelectric materials, nanostructuring techniques to improve their performance, and efficient energy conversion circuitry to harvest and store the produced power are all key technologies in PENGs. The output voltage, current, and power density of PENGs are performance factors that influence their efficacy in harvesting energy from tiny mechanical motions. Furthermore, mechanical sensitivity, energy conversion efficiency, and durability are critical for enhancing their functioning in a variety of applications. PENGs have received a lot of interest because of their potential to power small electronic devices, sensors, and even contribute to energy harvesting from ordinary motions, opening the way for future self‐powered, low‐maintenance systems. These PENGs can be applied to the unplanar and dynamic human body surface as they are made from flexible piezoelectric thin films.^[^ ^52^ ^]^ They have been included in flexible monitoring systems and implantable devices to generate energy from routine human actions (i.e., walking, bending joints, or even the pulsing of blood vessels). In this regard, PENGs can be one of the energy harvesters that are widely used for biomedical applications without external batteries or frequent recharging. Incorporating PENGs into wearable bioelectronics offers enormous potential for continuous health monitoring. For example, these nanogenerators could be included in flexible electronic patches to gather energy from body motions and use it to power sensors that track vital indications like heart rate, breathing, or neuromuscular activity. PENGs are also helpful in overcoming the difficulties of power supply use in implanted medical devices. A dependable and long‐lasting power supply is necessary for surgical implants like pacemakers, neurostimulators, or medication delivery devices. These devices can function independently and require fewer battery changes or external connections when nanogenerators using the body's mechanical energy are integrated into them. This might increase patient comfort, minimize surgical procedures, and increase the overall dependability and safety of the implants.

**Figure 3:**
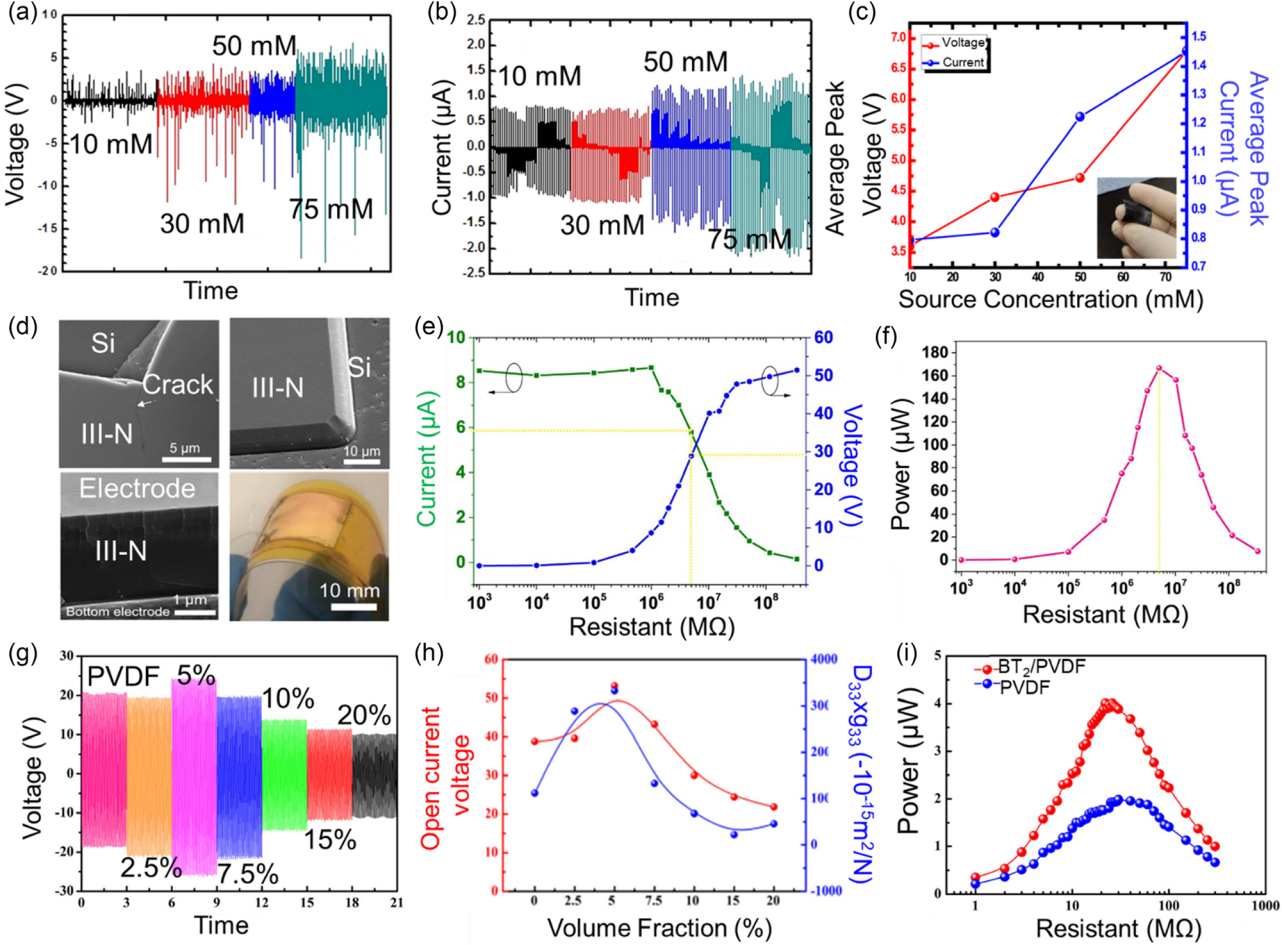
Inorganic piezoelectrical nanogenerators. a) Output voltage and b) current from the ZnO‐nanorod‐based PENGs on carbon paper with various molar concentrations measured with a strain frequency of 2 Hz. c) Average peak voltage and current outputs of the PENGs with varying molar concentrations from 10–70 mm. The inset shows the flexibility of the PENG device. (a–c) Reproduced with permission.^[^ ^59^ ^]^ Copyright 2017, IOP Publishing Ltd. d) i–iii) Scanning electron microscopy (SEM) images of: (i) thin‐film edge of the as‐diced sample, (ii) thin‐film edge after removal of microcracks, and (iii) cross section of the flexible piezoelectric generator (F‐PEG). iv) Digital image of the F‐PEG bent by hand. e,f) Experimental results of current and voltage (e) and of power (f) change with load resistance at maximum compression of 15 mm and a compressing time of 0.08 s. e,f) Reproduced with permission.^[^ ^62^ ^]^ Copyright 2019, Elsevier. g) Voltage of the BT2/PVDF FPEHs with varying BT2 content under 3 g acceleration. h) Open‐circuit voltage (peak‐to‐peak) and *d*_33_ × *g*_33_ values for BT2/PVDF textured composites with varying BT2 content. i) Power generation of the PVDF and 5 vol% BT2/PVDF FPEHs with varying external load resistance. g–i) Reproduced with permission.^[^ ^76^ ^]^ Copyright 2018, Elsevier.

Generally, inorganic materials have been used for piezoelectric devices with the advantages in terms of a high electromechanical coupling factor, high Curie temperature, and piezoelectric constant. They have been widely utilized as energy harvesters. Inorganic piezoelectric materials are naturally brittle, limiting their use in flexible PENGs. As a result, they are typically used in flexible organic matrixes as piezoelectric fillers. In 2006, Wang et al. proposed a wurtzite‐zinc oxide (ZnO)‐based PENG.^[^ ^53^ ^]^ As a traditional semiconductor, ZnO has highly diverse nanostructures and design abilities, so it can be used to make flexible PENG devices with various topologies.^[^ ^54^, ^55^ ^]^ It also has a low piezoelectric constant, a low dielectric constant, and a moderate piezoelectric strain constant. Consequently, such ZnO‐based PENGs can act as a platform that supplies a high piezoelectric voltage constant in various nanostructures.^[^ ^56^, ^57^, ^58^ ^]^ For example, Banna et al. reported a ZnO‐based PENG which was grown on flexible carbon paper, exhibiting an output open voltage (*V*_oc_: 3.6–6.8 V) and output short current (*I*_sc_: 0.79–1.45 μA) (**Figure** **3**a–c).^[^ ^59^ ^]^ The ZnO nanorod‐based PENG provides a controllable and enhanced output power in a flexible self‐powering system. For biocompatibility, Group‐III‐N (III‐N) materials with wurtzite structures, such as aluminum nitride (AlN), gallium nitride (GaN), and aluminum gallium nitride (Al, Ga)N, have been recognized in the PENGs.^[^ ^60^, ^61^ ^]^ Such PENGs were fabricated with a multilayer III‐N thin film that included an AlN as the buffer layer, an Al_*x*_Ga_1−*x*_N as the interlayer, and a GaN as the top layer (Figure 3d).^[^ ^62^ ^]^ The resulting PENG generated a *V*_oc_ of 50 V, an *I*_sc_ of ≈15 μA, a power density of up to 167 μW, and high stability for more than 30 000 cycles at a load resistance (5 MΩ) (Figure 3e–f).

Perovskite‐structured piezoelectric and wurtzite‐structured materials have been adopted in commercial applications to improve piezoelectric performance.^[^ ^63^, ^64^, ^65^, ^66^, ^67^, ^68^, ^69^, ^70^, ^71^, ^72^ ^]^ The most common piezoelectric components for flexible PENGs are perovskite‐structured materials.^[^ ^73^, ^74^ ^]^ For example, the piezoelectric particles were well distributed in solid silicone rubber for fabricating a stretchable PENG, exhibiting exceptional mechanical (30% of stretchability) and electrical properties (≈81.25 μW cm^−3^ of volume power density).^[^ ^75^ ^]^ Fu et al. reported a textured nanocomposite‐based flexible PENG device by the incorporation of piezoelectric BaTi_2_O_5_ nanorods into a poly(vinylidene fluoride) (PVDF) matrix (Figure 3g–i).^[^ ^76^ ^]^ This PENG demonstrated an outstanding output performance, including around 53.2 V of *V*_oc_, a high volume power density (≈27.4 μW cm^−3^), and a long‐term operating lifetime that displayed a consistent and reliable performance over 330 000 cycles.

**Figure 4:**
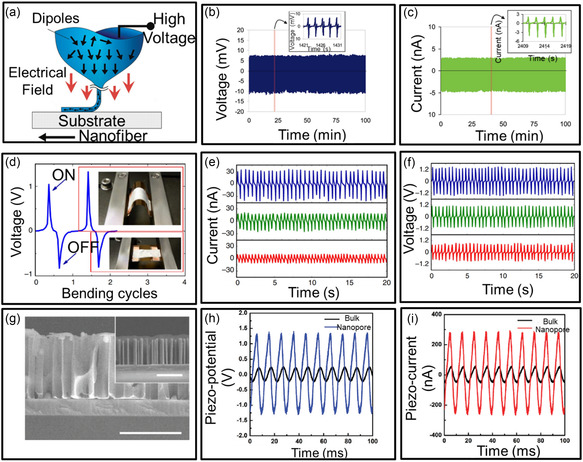
Organic PENGs. a) Near‐field electrospinning combining direct‐write, mechanical stretching, and in situ electrical poling to create and place PENGs onto a substrate. b) Output voltage and c) output current of a PVDF nanogenerator subject to different stretch–release cycling frequencies of 2–4 Hz. a–c) Reproduced with permission.^[^ ^82^ ^]^ Copyright 2010, American Chemical Society. d) Measured voltage response of an array of P(VDF‐TrFe) fibers under cycling bending at 1 Hz. The top and bottom insets show photographs of the device during bending and release, respectively. e,f) Measured short‐circuit output current (e) and voltage (f) under dynamic bending test at 2 Hz. d–f) Reproduced under the terms of the CC‐BY Creative Commons Attribution 4.0 International license (https://creativecommons.org/licenses/by/4.0).^[^ ^83^ ^]^ Copyright 2013, Springer Nature. g) Cross‐sectional SEM images of the porous PVDF nanostructure using a ZnO nanowire. The inset shows a PVDF nanostructure before removing ZnO nanowires. (Scale bar: 5 μm). h) Piezoelectric potential and i) piezoelectric currents from the porous PVDF and bulk structure under the same force. g–i) Reproduced with permission.^[^ ^84^ ^]^ Copyright 2011, American Chemical Society.

Organic piezoelectric materials (e.g., PVDF or its copolymer‐based composites) have a lower piezoelectric coefficient than inorganic piezoelectric materials.^[^ ^77^, ^78^ ^]^ However, natural flexible and lightweight organic piezoelectric materials are appropriate for wearable PENG devices.^[^ ^79^, ^80^, ^81^ ^]^ In addition, using inorganic and organic filler materials together can provide additional nucleation sites to encourage the piezoelectric‐phase PVDF formation, improving the PVDF‐based PENG devices’ output performance substantially. Furthermore, PVDF might be readily molded into various geometries owing to its polymer nature, including porous membranes, hollow nanofibers, ultrathin membranes, and nanofibers. For example, Lin et al. reported a directly written piezoelectric polymeric nanogenerator on flexible plastic substrates using near‐field electrospinning (**Figure** **4**a–c).^[^ ^82^ ^]^ The PVDF nanofibers were prepared to utilize an electrical poling process and in situ mechanical stretch at a high bias voltage (>10 kV). Under multiple long‐term reliability tests, a PVDF nanofiber‐based device has an output performance with a current of 0.5–3 nA and voltage of 5–30 mV. To fabricate a vibration‐driven generator, researchers attempted to sandwich randomly oriented electrospun PVDF nanofibers between two plate electrodes. Persano et al. placed electrospun piezoelectric P(VDF‐TrFE) fibers onto a rotating collector to form a large‐area, flexible, free‐standing film (Figure 4d–f).^[^ ^83^ ^]^ In order to obtain sufficient fiber stretching for effective energy conversion, the aligned PVDF fibers were extensively placed on two electrodes affixed to a flexible substrate. The aligned P(VDF‐TrEE) fibers exhibited a stable output current and voltage during a bending test of up to 1000 cycles. Cha et al. proposed a template‐assisted nanoporous PVDF structure, allowing a high piezoelectric efficiency (Figure 4g).^[^ ^84^ ^]^ The output voltage and current generated from porous PVDF nanogenerators were 2.6 V and 0.6 μA, 5.2 times and 6 times higher than those from bulk PVDF nanogenerators, respectively (Figure 4h–i). This porous PVDF nanogenerator produced a power density of 0.17 mW cm^−3^. The proof‐of‐concept results suggest they could be used in highly efficient energy‐harvesting devices. Thus, they exhibit promising potential as a core component of self‐powered wearable devices in wireless human healthcare monitoring and management.

#### 2.2.2 Triboelectric Nanogenerators

**Figure 5:**
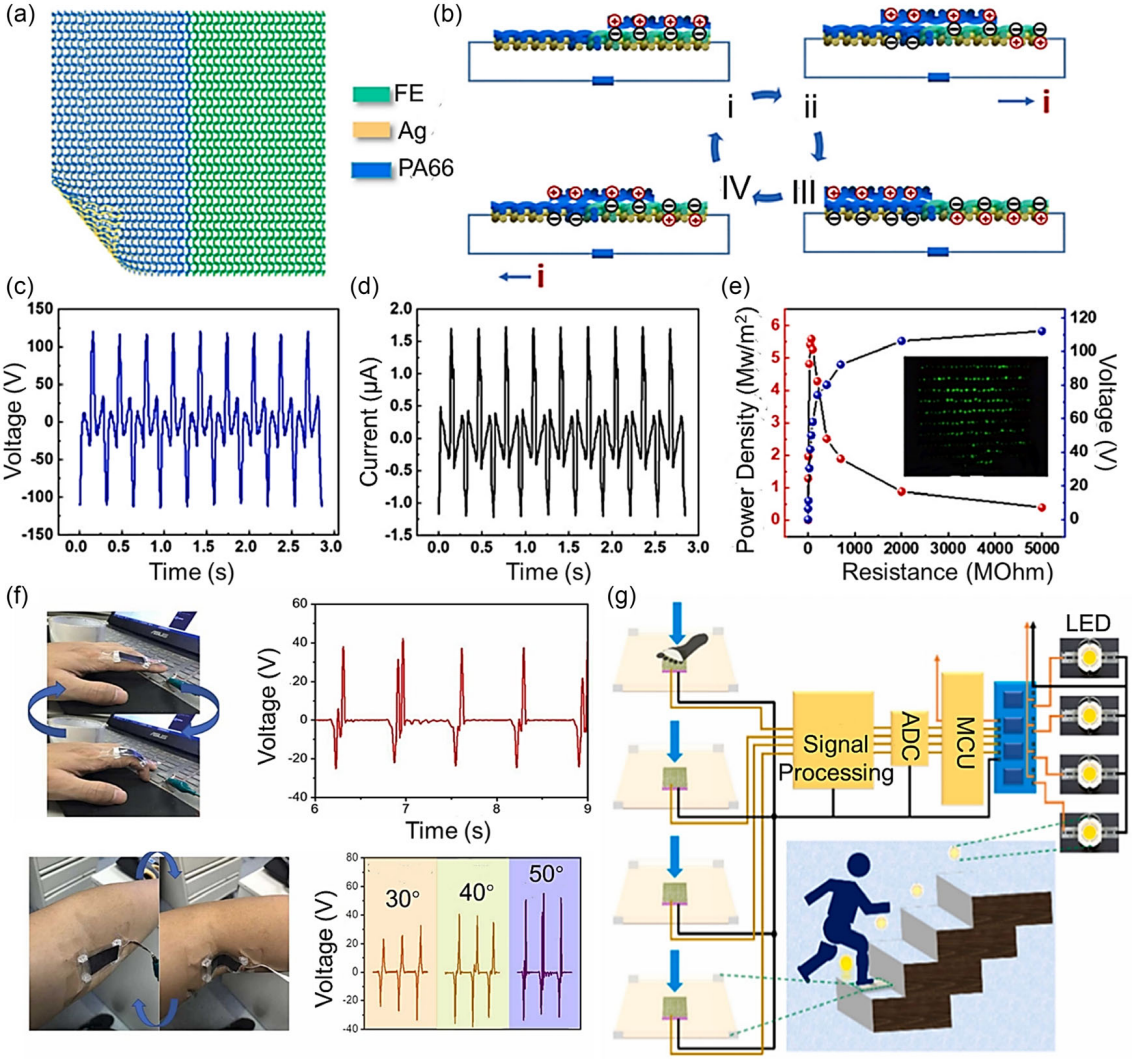
Triboelectric energy nanogenerators for human motion harvester. a) Schematic illustration of the TENG. b) Working mechanism of the TENG. c) Output open‐circuit voltage and d) short‐circuit current of the textile TENG at a motion frequency of 3 Hz. e) Output power density and voltage of textile TENG with various external load resistances (0–2000 MΩ). The inset photograph shows that the textile TENG lit 250 LEDs. a–e) Reproduced with permission.^[^ ^104^ ^]^ Copyright 2021, Elsevier. f) Voltage signal of the *M*‐TENG in response to the continuous bend of the (upper) finger and (lower) elbow. Reproduced with permission.^[^ ^90^ ^]^ Copyright 2022, Elsevier. g) Schematic illustration of the TENG utilized as a self‐powered footstep motion sensor that automatically controls the light in the stairs based on human foot motions. Reproduced with permission.^[^ ^105^ ^]^ Copyright 2021, Elsevier.

The TENG is a revolutionary, cutting‐edge energy collection system that utilizes electrostatic induction and contact electrification to convert ubiquitous mechanical energy into electricity. The term “triboelectric” describes the transfer of electrons from one surface to another by contact electrification and electrostatic induction.^[^ ^85^ ^]^ Electrons remain within the contacted surfaces, and when the surfaces are separated, one surface becomes electronegatively charged, and the other becomes electropositive. Charge transferring is then shunted through the charge trapping and collection layer during the next contact‐separation cycle and transferred to the charge storage layer for further use (**Figure** **5**a,b). TENG performance characteristics include a number of essential aspects. These characteristics include output voltage, current, and power density, which determine their capacity to transform mechanical energy into electrical power. Furthermore, parameters like as contact area, material selection, and the frequency and amplitude of mechanical vibrations all have a substantial impact on TENG efficiency. These devices have gained popularity because of their capacity to harvest energy from a variety of mechanical movements, making them a potential alternative for self‐powered electronics, wearable technology, and energy‐efficient sensors in a variety of applications. TENGs, given their selective sensitivity to dynamic pressures, are ideal for controlling various electrical devices and simulating the actions of fast‐adapting receptors.^[^ ^85^ ^]^ Their self‐powered applications are made possible by the extra benefit of generating energy during mechanical stimulation.^[^ ^86^, ^87^, ^88^, ^89^ ^]^ TENGs have been employed in various applications, including self‐powered active sensors, electrochemical treatments, wearable sensors, and mechanical energy harvesting.^[^ ^90^, ^91^, ^92^ ^]^ Compared to other energy converters, they can be designed as single or dual electrodes with ultra‐lightweight and flexibility.^[^ ^93^, ^94^, ^95^, ^96^, ^97^, ^98^ ^]^ These distinguishing features enable TENG‐based electronic devices to offer self‐powering ability, dependability, cost‐effectiveness, and multifunctionality, enabling them especially appropriate to construct inconspicuous smart flooring systems in a world where such systems will become ubiquitous. It is essential to conduct an in‐depth review of this topic in order to identify the issues that must be addressed in the future for continued growth.

Recently, TENGs woven into textiles for capturing the energy generated by human motion have attracted considerable interest. Exceedingly precise and optimized textile TENG designs have been developed to harvest energy from human activities.^[^ ^99^, ^100^, ^101^ ^]^ Zhou fabricated a woven‐structured TENG (W‐TENG) that can effectively capture and transform human body motions into electrical power.^[^ ^102^ ^]^ To fabricate the W‐TENG, two opposing triboelectric materials (polyester and nylon) were woven with an Ag cloth that served as a conductor. Charge transfer between the textiles promotes electrical energy generation when the structure is stretched or compressed. Dong et al. successfully incorporated a textile TENG into a wearable knee joint, producing a maximum output of 60 V.^[^ ^103^ ^]^ The textile TENG was composed of triboelectric components that were woven around a conducting cloth. Then, these threads were knitted together to form a patch on a pair of pants’ knee joints. Interestingly, it was discovered in the same study that the output of the textile TENG increased after the washing process. Xu et al. fabricated a textile TENG that produced an output voltage of 232 V and was verified to power small electronic devices effectively (Figure 5c–e).^[^ ^104^ ^]^ Two fabric materials were knitted onto an Ag cloth to form the textile TENG. A power management module was subsequently added to the device, enabling it to power small electronic devices successfully by converting the textile TENG's AC output to DC and stabilizing its high voltage and low current.

In 2022, Yi et al. reported an integrated, TENG‐based wearable sensing device that can monitor human motions in real time (Figure 5f).^[^ ^90^ ^]^ Using the MXene, the self‐powered TENG showed a power output (≈816.6 mW m^−2^), and it could be easily attached to the human body using a skin‐like flexible styrene–ethylene–butylene–styrene substrate. The TENG could harvest power from finger motions and arm bending. Based on the results, these devices have been boosting the enormous potential for wearable health monitoring devices. As another application of TENG, Bhatta et al. developed a self‐powered motion sensor that can automate stair lighting based on the movement of a person's foot up a stairway by fabricating TENG‐based composite nanofibers that can harvest energy and operate power (Figure 5g).^[^ ^105^ ^]^ The TENG‐based composite nanofibers generated a 4.6 mW of power and 11.213 W m^−2^ of power density, which is sufficient to operate low‐power electronics such as sports watches, thermoshygrometer sensors, and hundreds of commercial light‐emitting diodes (LEDs). Furthermore, it showed high stability through 60 000 cycles of repeated contact separation actions. Validations of an automatic stair lighting control application by sensing human movements on a stairway exhibited their high‐performance energy scavenging and self‐powered monitoring capabilities (Figure 5h).

**Figure 6:**
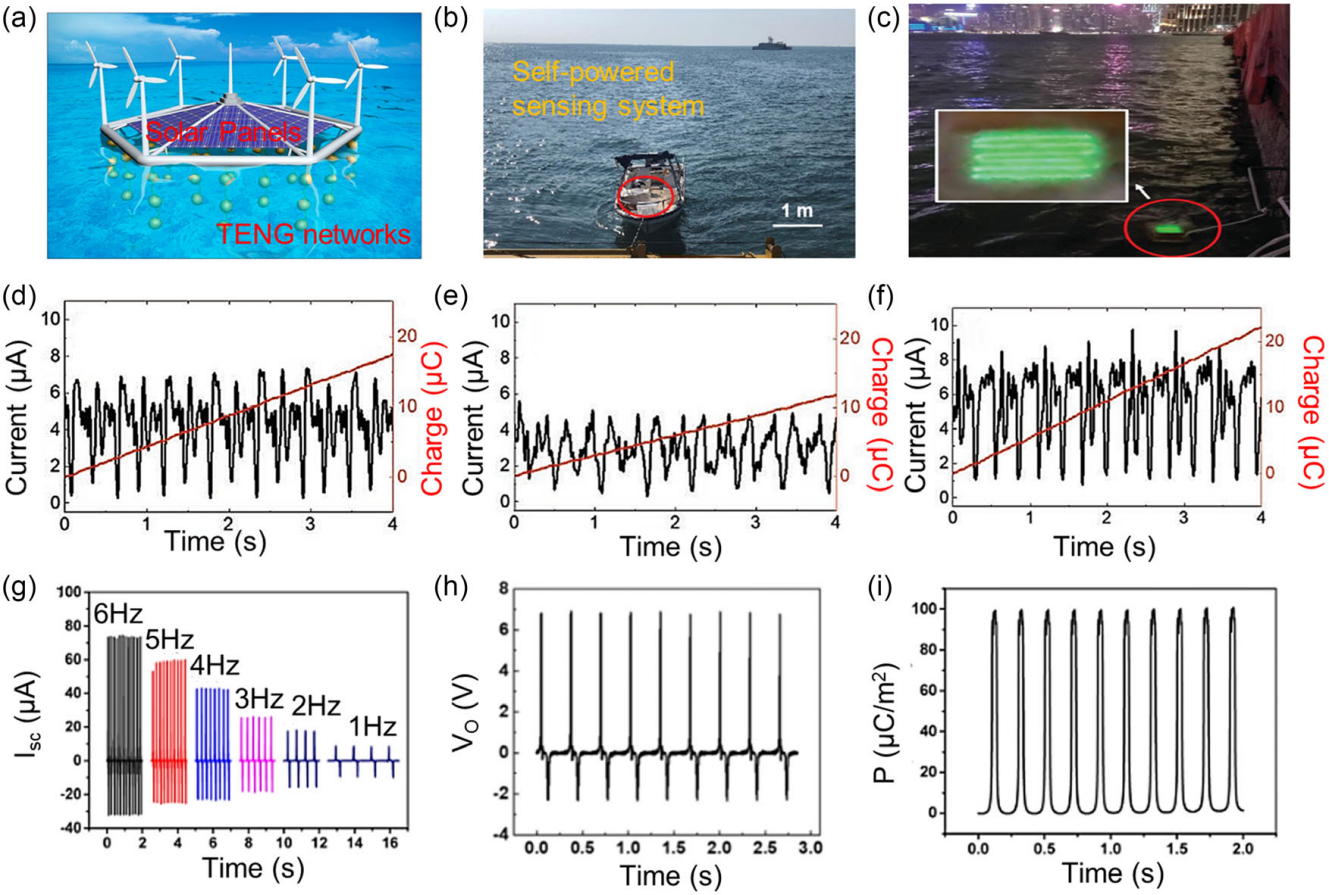
Triboelectric energy nanogenerators for environmental energy harvester. a) A blue energy dream through 3D networks of TENGs, wind generators, and solar panels can be installed above the water surface to add power and maximize space utilization efficiency. Reproduced with permission.^[^ ^107^ ^]^ Copyright 2017, Elsevier. b) Self‐powered wireless sensing nodes transmission system enabled by the hybridized nanogenerator in Bo Hai. Reproduced with permission.^[^ ^108^ ^]^ Copyright 2020, Elsevier. c) 150 LEDs were powered by water waves (in Victoria Harbor in Hong Kong). d–f) The output current and transferred charges of the WT‐TENG box are driven in the horizontal linear mode in the *x*‐direction (d), the *y*‐direction (e), and 45° to the *x*‐ and *y*‐direction (f). c–f) Reproduced with permission.^[^ ^110^ ^]^ Copyright 2021, Wiley‐VCH. g–i) The output performance of LP‐PLL TENG, including: (g) *V*_o_ under various driven frequencies, (h) *V*_o_ under 5 Hz contact frequency, and (i) current density. g–i) Reproduced with permission.^[^ ^116^ ^]^ Copyright 2019, Elsevier.

Ocean wave energy is plentiful and has enormous potential for harnessing sustainable energy. In recent years, substantial progress has been made in harnessing power from blue energy (i.e., water wave) using the TENG operating principle (**Figure** **6**a,b).^[^ ^106^, ^107^, ^108^ ^]^ Chen et al. demonstrated the use of a TENG network to collect electrical energy from large‐scale water wave energy.^[^ ^109^ ^]^ High‐load and random oscillatory motions were used in this TENG gadget to create contact and separation. Wu et al. demonstrated a water‐tube‐based TENG device that is compact, modular, and high‐performing in low‐frequency and irregular movements (Figure 6c–f).^[^ ^110^ ^]^ The TENG system was prepared by wrapping copper tapes with deionized water encapsulated around the exterior of a fluorinated ethylene‐propylene tube. It showed the highest output voltage (223 V) and charge (73 nC) from a finger‐sized single tube‐based TENG. Xia et al. reported a water‐balloon TENG with 28 times the efficiency of conventional double‐stacked TENGs for harvesting energy from low‐frequency ocean waves.^[^ ^111^ ^]^ Wang et al. created a pear‐shaped hybrid generator to harness the power of unpredictable low‐frequency water waves.^[^ ^112^ ^]^ This hybrid TENG functions by pushing and rolling a magnetic cylinder with the water action. Jiang et al. emphasized the importance of mechanical design in the wave TENG system.^[^ ^113^ ^]^ It was found that the effective wave frequency of TENG devices can be increased from low levels obtained from waves by adding appropriate stiffness springs; this is known as a “spring‐assisted TENG”. This resulted in a 150% improvement in converted electrical energy or efficiency and a 113% increase in collected charge.

The scientific community is increasingly interested in wind energy extraction as a clean, efficient energy source. In 2018, more than 5% of the world's power came from wind energy.^[^ ^114^ ^]^ Electricity is often produced from wind using turbines with electromagnetic generators. Researchers have recently attempted to use TENG technology to generate electricity from wind or airflow energy.^[^ ^115^, ^116^, ^117^ ^]^ Chen et al. reported a Bernoulli‐effect‐based TENG device that operates at low wind speeds with high efficiency.^[^ ^118^ ^]^ It comprises two four‐mode triboelectric laminated sheets in contact. This TENG device's remarkable sensitivity allowed it to produce power from a slight wind or a human's swinging arm. Yang et al. demonstrated a dual‐TENG generator to capture wind energy and quantify wind speed and direction.^[^ ^119^ ^]^ This TENG system is unique because it uses wind‐induced resonance vibration to generate output (100 V of output voltage and 1.6 μA of output current) by sandwiching a fluorinated ethylene–propylene film between two aluminum foils. Feng et al. reported a simple and affordable TENG tree system based on biodegradable plant leaves for generating energy from wind (Figure 6g–i).^[^ ^116^ ^]^ Thus, TENGs could power an electrical watch with high output (1000 V of voltage and 60 μA of short‐circuit current). Zhang et al. reported an innovative hybrid TENG generator that could capture low‐speed wind energy. To implement the contact‐separation mode of the TENG gadget, they used a rotating motion activated by airflow.^[^ ^120^ ^]^ TENG applications have multiplied as various TENG devices with increased efficiency and output have been developed. However, four major barriers prevent the commercial implementation of TENGs for widespread and long‐term energy generation: electrical output, environmental stability, industrialization, and standardization.

### 2.3 Radio Frequency Energy Harvesters

**Figure 7:**
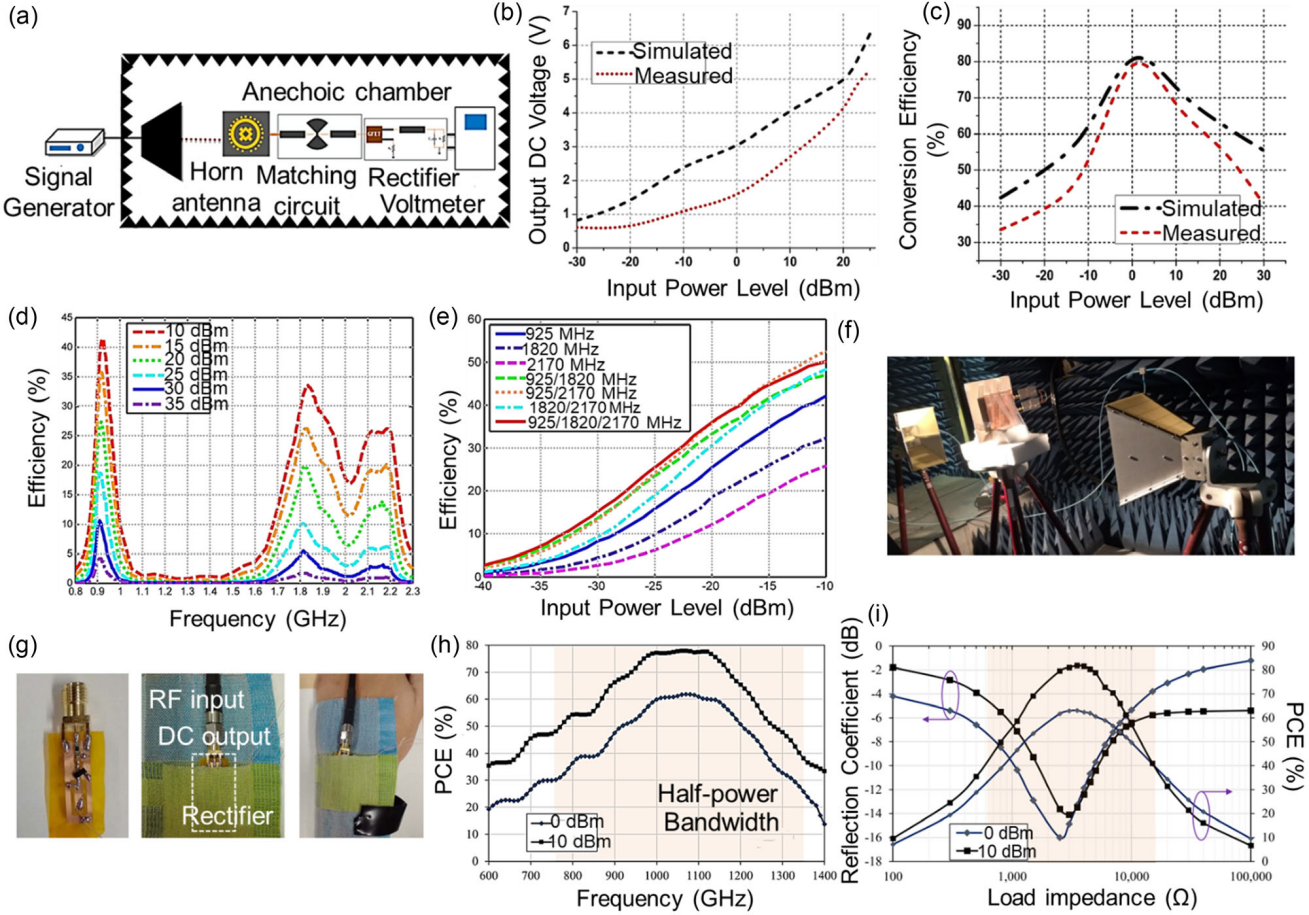
RF energy harvesting. a) Illustration shows an experimental setup of graphene rectenna inside an anechoic chamber. b) Variation of output DC voltage and input power level. c) Simulated and measured conversion efficiency of the rectenna. a–c) Reproduced with permission.^[^ ^124^ ^]^ Copyright 2020, Elsevier. d) Photograph of rectenna measurement in an anechoic chamber. e) Measured RF‐to‐DC efficiency of the rectifier versus frequency at various input RF power levels. f) Measured RF‐to‐DC efficiency of the rectifier for various single‐tone and multitone input signals. d–f) Reproduced with permission.^[^ ^126^ ^]^ Copyright 2017, IEEE. g) Photographs of the connectorized rectifier test setup. h) Measured PCE of the rectifier over a frequency sweep showing a 57% half‐power 3 dB fractional bandwidth. i) Measured reflection coefficient (S11) of the rectifier for varying load impedances at 1 GHz; the shaded region indicates the half‐power load range. g–i) Reproduced with permission.^[^ ^128^ ^]^ Copyright 2021, IEEE.

In the 20th century, RF was invented, and later attempts have been made to transform it into a DC signal.^[^ ^121^ ^]^ A rectenna relies on an antenna to receive RF energy and convert it into usable power for electronic devices.^[^ ^122^ ^]^ An optimized dual‐ or triple‐band has been established for powering devices. Available operating bands include Wi‐Fi, GSM 900, GSM 1800, and millimeter waves with a frequency range from 30 GHz to 300 GHz.^[^ ^123^ ^]^ Antennas and their components, including DC pass filters, rectifiers, and impedance‐matching circuits, are displayed in **Figure** **7**a–c.^[^ ^124^ ^]^ The DC pass filter smoothens the output and eliminates rectifier harmonics by acting as a low‐pass filter. The converted DC voltage is stored in the final block's storage device. The size and structure of the antenna determine its power capacity.^[^ ^125^ ^]^

Shen et al. reported a dual‐port, triple‐band rectenna for RF energy harvesting (Figure 7d–f).^[^ ^126^ ^]^ The reception antenna can operate in the GSM‐1800, UMTS‐2100, and GSM‐900 bands. The RF harvester exhibited more than 40% efficiency, 600 mV output voltage, and 500 μW cm^−2^ power density. Recently, a rectenna array was integrated into wearable textiles. The RF‐to‐DC conversion efficiency for the rectifier system was 70%.^[^ ^127^ ^]^ The gathered power measured at 60 cm from the RF source was 80 μW. There was no power management circuit employed in the proposed system. Instead, the RF energy in the environment was amplified before being gathered. The system's ability to power three LEDs at a distance (60 cm) was confirmed during testing, proving that the recommended system can also charge supercapacitors or power sensors. A textile supercapacitor is proposed as the storage element of a textile‐based RF energy harvester. With an RF‐DC efficiency of 80%, the rectenna can charge a supercapacitor to 1.5 V in 4 min at a distance of 4.2 m from the source without power management circuitry (Figure 7g–i).^[^ ^128^ ^]^ In 2015, a wireless optogenetics system based on an adaptable RF harvester was reported.^[^ ^129^ ^]^ A stretchable antenna was implanted inside a mouse model. The device could control spinal pain circuits and endure mechanical deformations. A wireless communication system with 15 μW cm^−2^ power density was achieved using a trehalose/oxygen BFC implanted in a month.^[^ ^130^ ^]^ Based on a study of RF energy harvesting, the 3D‐printing technique has been developed for constructing the flexible antenna owing to their advantages, such as being lightweight, cost‐effective, and scalability.^[^ ^90^, ^131^ ^]^

### 2.4 Solar Cells

Solar energy, as a sustainable green energy, is a clean, renewable, and abundant energy source with a wide range of applications. One interesting area is incorporating solar cells into flexible bioelectronic devices, solar cells are devices that convert sunlight directly into energy. Traditional solar cells are rigid and large, making them difficult to incorporate into flexible bioelectronic systems. However, recent advances in materials science and engineering have resulted in flexible, semitransparent, and biocompatible solar cells that can be easily integrated into wearable devices, implants, and biological tissues. Numerous solar cell materials, including perovskite and dye‐sensitized materials, have been explored for gathering light energy for powering intelligent wearable devices.^[^ ^132^ ^]^

**Figure 8:**
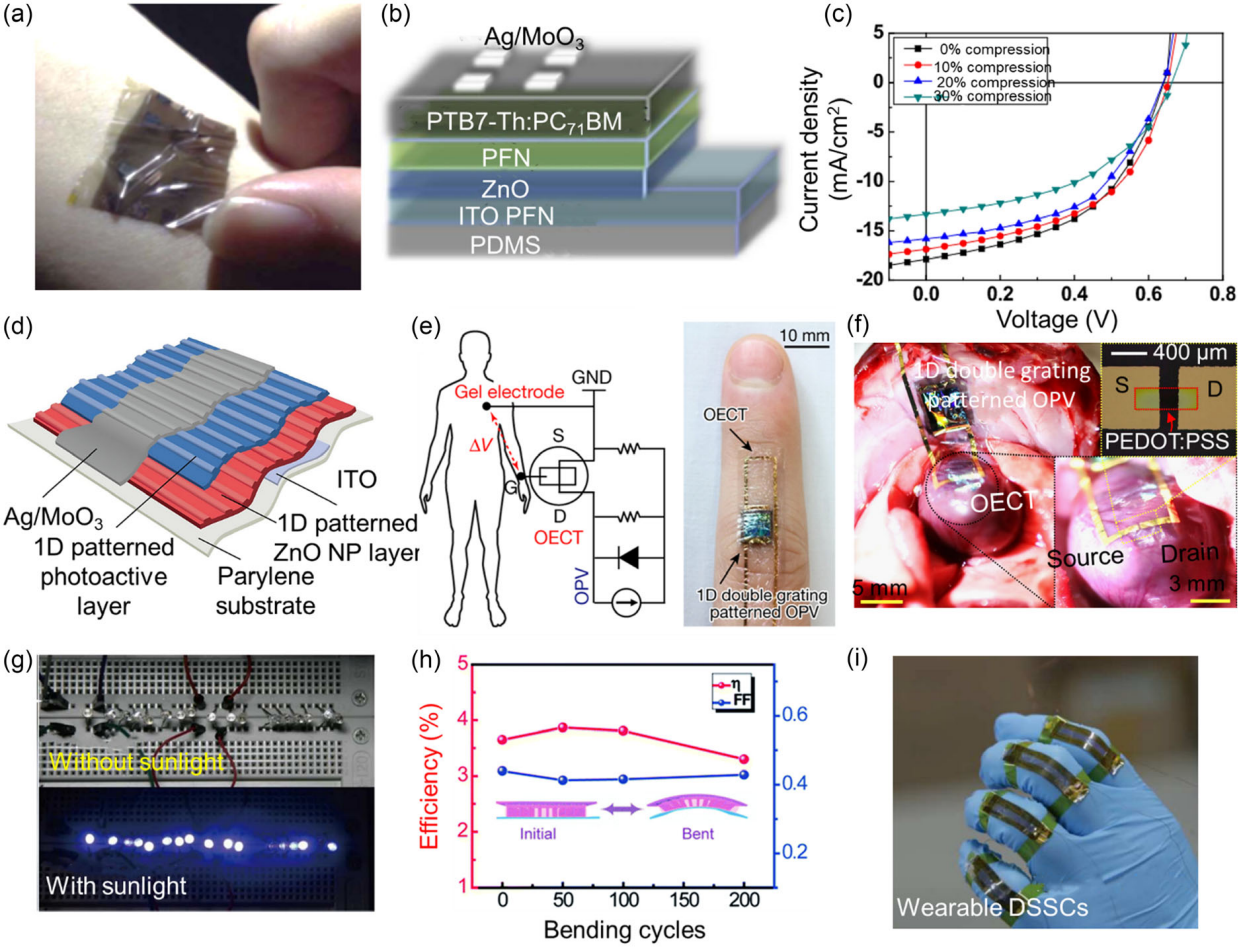
Flexible OSCs. a) Fabricated stretchable device attached to the skin. b) Device configuration of the fabricated solar cell device. c) Current density–voltage characteristics of the studied solar cell devices on a prestrained (100%) 3M elastomeric tape under various compressed conditions. a–c) Reproduced with permission.^[^ ^137^ ^]^ Copyright 2018, Elsevier. d) Structure of the flexible OSC device. e) Wiring diagram for cardiac signal recording and photograph of the self‐powered integrated electronic device attached to a finger. f) Photograph of the self‐powered integrated electronic device attached to the heart of a rat (left) and enlarged images of the channel area ((a) right, top) and these source‐drain electrodes ((a) right, bottom). d–f) Reproduced with permission.^[^ ^138^ ^]^ Copyright 2018, Springer Nature. g) Photographs of driving LED measurements using foldable DSSCs. h) Stability measurements of the flexible DSSC after periodic bending. i) Photograph of the wearable DSSCs affixed to human fingers. g–i) Reproduced with permission.^[^ ^145^ ^]^ Copyright 2015, Royal Society of Chemistry.

Organic solar cells (OSCs) have immense potential for indoor applications given their low cost, superior mechanical flexibility, low thickness, and advantageous optical energy bandgap.^[^ ^133^ ^]^ In addition, they possess various advantages, including low weight, mobility, flexibility, semitransparency, low processing temperatures, and cost‐effectiveness.^[^ ^134^ ^]^ To be suitable to be combined with wearable electronics, OSCs must exhibit high elasticity, mechanical stability, and transparency.^[^ ^135^ ^]^ Shin et al. developed a transparent and flexible graphene electrode doped with graphene quantum dots and connected with silver nanowires.^[^ ^136^ ^]^ The resistance of the graphene–AgNWs electrode was decreased with the increase of the concentration of graphene quantum dots. The power conversion efficiency (PCE) was improved by up to 3.66%, and the flexibility of the electrode was confirmed through 1000 cycles of bending tests. Hsieh et al. reported a flexible and skin‐attachable OSC using a buckle‐on‐elastomer strategy (**Figure** **8**a–c).^[^ ^137^ ^]^ They combined an ultrathin PEN sheet with a prestretched commercial (3M) elastomer to form a flexible and highly effective OSC. Owing to the prestrained conditions of the elastomer component, the residual strain inside the elastomer produced a wrinkled substrate. The results showed that the OSC's PCE increased up to 5.61%, while the PCE sustained around 74% of its overall value at 30% compression and 64.3% of its effectiveness through 50 cycles of compression–stretching (0–30%). Park et al. constructed double‐grating OSCs on a thin substrate (Figure 8d–f).^[^ ^138^ ^]^ The OSCs that were exhibited not only had highly flexible external surfaces and a high power (11.46 W g^−1^) but also a high PCE of 10.5%. The authors additionally paired OSCs with organic electrochemical transistors to construct self‐powered and flexible electrical devices. When applied to the skin or other tissues, they showed that self‐powered electrical devices can detect vital signs with a high signal‐to‐noise ratio.

Dye‐sensitized solar cell (DSSC) is the most widely used battery device due to its scalable roll‐to‐roll manufacturing process, good mechanical stability, and low‐cost and environmental‐friendly materials.^[^ ^139^, ^140^ ^]^ DSSCs can be highly flexible, enabling them good candidates for developing wearable power sources. Specifically, flexible DSSCs on polymer substrates coated with a transparent conductive layer have been widely manufactured.^[^ ^141^, ^142^, ^143^ ^]^ Xu et al. employed a colloidal crackle pattern to fabricate a large‐area flexible transparent conductive CuS sheet as a counter electrode to assemble flexible DSSCs.^[^ ^144^ ^]^ The resulting flexible transparent DSSCs exhibited a PCE of 4.54%. The device retained over 90% of its original PCE after 500 bending cycles. Similarly, Zhou et al. reported a highly flexible DSSC based on a transparent Pt nanowire‐based counter electrode (Figure 8g–i).^[^ ^145^ ^]^ Such Pt nanowire electrodes provided high mechanical elasticity after 200 bending cycles, and the final DSSC could have a PCE of 3.82% and maintain over 90% of PCE after the bending test. In addition, the DSSC could be attached to human fingertips with excellent functional stabilities.

**Figure 9:**
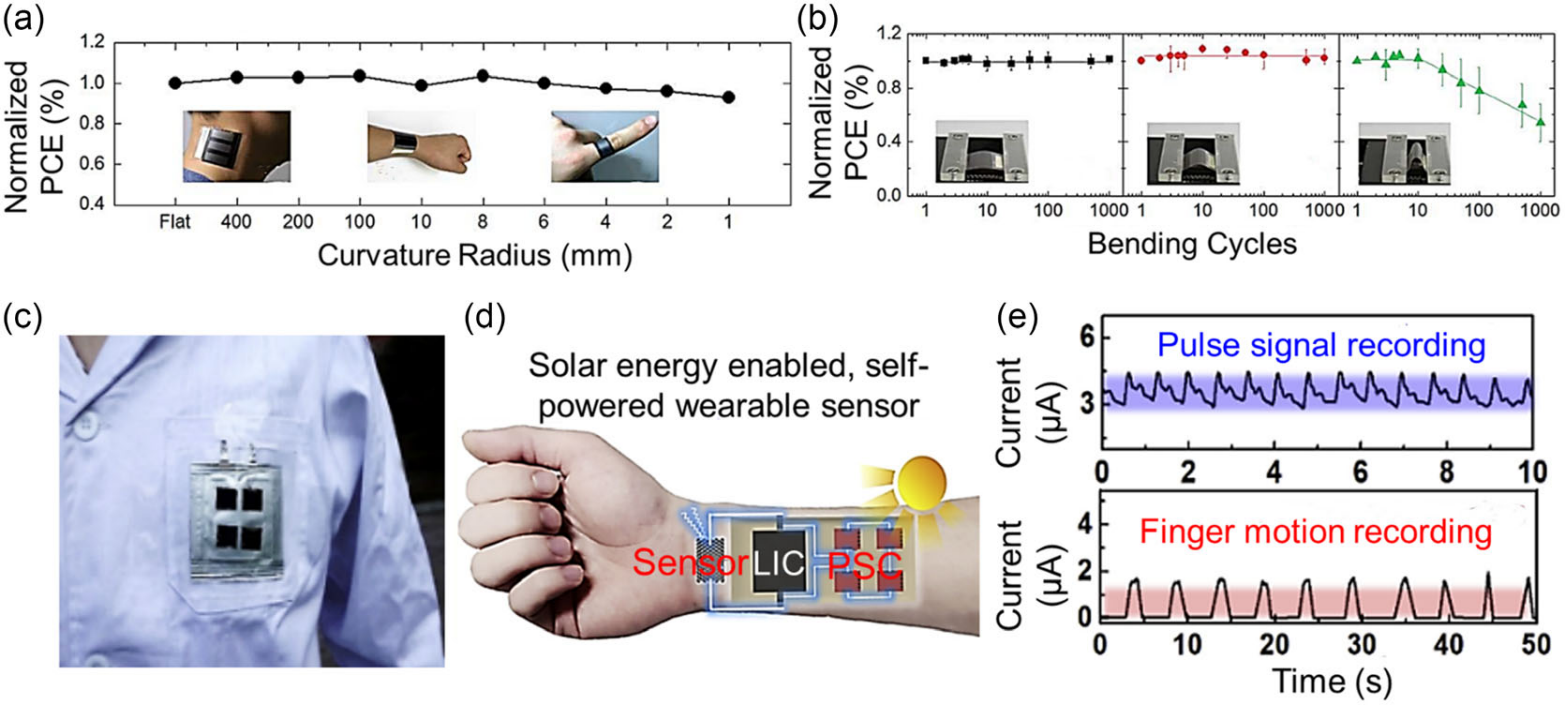
Flexible PSCs. a) Normalized PCE measured after bending the substrate within a specified radius from 400 to 1 mm. The inset shows the photos of sensors attached to the human neck, wrist, and finger corresponding to 400, 10, and 4 nm bending radii, respectively. b) Normalized PCE of flexible perovskite devices as a function of bending cycles with different radii of 400, 10, and 4 mm. The inset shows the real images taken during the bending test. a,b) Reproduced with permission.^[^ ^148^ ^]^ Copyright 2015, Royal Society of Chemistry. c) Photograph of the as‐integrated flexible PSC–lithium‐ion capacitor device, showing good device flexibility. d) Measured output current from the pulse signal (upper panel) and finger motion (lower panel) under light illumination. e) Schematic diagram of a solar energy‐enabled self‐powered wearable sensor. c–e) Reproduced with permission.^[^ ^149^ ^]^ Copyright 2019, Elsevier.

Photoactive perovskite has recently been used to fabricate flexible solar cell systems to power portable electronic devices. Compared to conventional solar cells, perovskite solar cells (PSCs) have various features, such as high electron transport efficiency. For example, Kaltenbrunner et al. developed a flexible PSC by incorporating a transparent polymer electrode and a chromium oxide–chromium interlayer. The resulting PSCs reach a PCE of 12% and a maximum specific power of 23 W g^−1^.^[^ ^146^ ^]^ Park et al. reported a wearable PSC using Li‐doped SnO_2_ as the electron transport layer.^[^ ^147^ ^]^ The flexible PSC demonstrated good adaptability with a high PCE of 14.78%. This flexible PSC can be attached to the wrist to power a small fan. Flexible PSCs with appropriate efficiency and light weight are ideal for self‐powered wearable devices. Kim et al. developed a flexible PSC based on a TiO_*x*_‐coated polyethylene naphthalate (PEN) substrate at low temperatures (**Figure** **9**a–b).^[^ ^148^ ^]^ It exhibited 12.2% PCE and constant performance up to 1000 bending cycles with different radii. This PCE could be attached to the human neck, wrist, and finger with no noticeable performance degradation. In order to further ensure sustainable powering, Li et al. reported a flexible PSC combined with a photorechargeable Li‐ion supercapacitor that enhances energy harvesting and storage efficiency (Figure 9c–e).^[^ ^149^ ^]^ With an overall PCE of 8.41%, the device could supply 3 V of output voltage and 0.1 A g^−1^ of discharge current density. In addition, even at high current densities of 1 A g^−1^, an overall PCE of over 6% could be achieved.

Overall, by integrating the abovementioned energy harvesters, wearable biomedical devices hold promise to assemble a self‐powered wearable system for digital healthcare. We will then introduce several representative wearable bioelectronic devices, enabling wireless communication without external batteries.

### 2.5 Incorporation of Liquid Metals in Energy Harvesters for Flexible Bioelectronics

Liquid‐metal‐based energy harvesters have recently gained considerable academic interest and discovered niche uses in the development of flexible bioelectronics due to their high electrical conductivity, flexibility, and biocompatibility.^[^ ^150^ ^]^ Finding a dependable and long‐lasting power supply is one of the main difficulties in maintaining these devices. Specifically, the flexibility of liquid metals enables the devices to conform to nonplanar and dynamic skin surfaces with minimal side effects,^[^ ^151^, ^152^, ^153^ ^]^ while self‐healing capabilities in some liquid metals can extend their applications under some harsh environment or during extended use.^[^ ^154^ ^]^ All these features enable liquid metal a strong contender to be applied in different wearable energy harvesters.

For instance, liquid metals can be included in triboelectric energy harvesters,^[^ ^155^ ^]^ which produce energy through the contact and separation of materials. For example, Zhang and colleagues improved the electrical performance of TENGs based on PVDF by incorporating Galinstan nanodroplets (liquid metal) into electrospun nanofibers made of PVDF*‐co*‐hexafluoropropylene (PVDF‐HFP). They utilized a PVDF‐HFP nanofiber membrane with 2% Galinstan as the negative tribolayer and thermoplastic polyurethane as the positive tribolayer. As a result, the TENG they developed achieved a peak open‐circuit voltage of 1680 V and a power density of 24 W m^−2^. These values represent a substantial improvement over the previous best‐performing PVDF‐based TENGs reported in the literature. Majidi and his research team have pioneered the development of an innovative elastomeric composite, incorporating sedimented liquid metal droplets, to create a TENG‐based energy harvesting system. This technology capitalizes on the precise assembly of liquid metal components to create distinct conductive and insulating regions within the composite material. This novel approach has yielded impressive results, including exceptional stretchability (able to withstand strains of over 500%), remarkable compliance resembling human skin (with a modulus of less than 60 kPa), outstanding device stability (demonstrating reliability over 10 000 cycles), and substantial electrical output performance (achieving a maximum peak power density of 1 mW cm^−^^2^). Moreover, the team envisions the integration of their sedimented liquid metal elastomer TENG (SLM‐TENG) with highly elastic and stretchable fabrics, thereby facilitating seamless incorporation into wearable electronics.

A soft wearable antenna plays a vital role in wearable electronic devices, enabling portable wireless functionality, wireless energy harvesting, and communication. Since the antenna's resonance frequency is determined by its inductive coil, which depends on factors such as resistance, inductance, and capacitance, it is essential for its circuit to exhibit excellent pattern ability, electromechanical properties with a low gauge factor, and minimal hysteresis. These characteristics are critical for matching the antenna's circuit to the desired frequency ranges.^[^ ^156^, ^157^ ^]^ An initial exploration of utilizing liquid metal technology in wireless wearable electronics was conducted by Alberto et al. They introduced an electronic tattoo that showcased various functions, including battery‐free wireless energy harvesting via a passive antenna for monitoring electrophysiological data. This unique tattoo pattern was printed onto tattoo paper using an ordinary desktop printer, utilizing Ag flakes ink. Subsequently, it underwent sintering with an EGaIn liquid metal alloy, rendering it both highly conductive and stretchable. The integration of inductive coil patterns and skin‐interfacing electrodes crafted from Ag–In–Ga enabled the creation of a wireless power transfer health‐monitoring system. Impressively, this system was capable of generating ≈300 milliwatts of power, falling well within the operational requirements of biomedical devices.^[^ ^158^ ^]^

Similarly, Lopes et al. introduced a novel composite material consisting of Ag microflakes, liquid metal microdroplets, and styrene–isoprene block copolymers, which they utilized for the development of digitally printed sensors, circuits, and antennas with exceptional deformability and wearability. This innovative 3D‐structured electronic material composite exhibits remarkable cohesiveness in a biphasic state, resulting in a stable electromechanical performance characterized by a low gauge factor of 0.9 and an impressive stretchability of up to 600%. The standout feature of this trinary composite, leveraging the unique properties of EGaIn, Ag flakes, and AgI_2_ microparticles, is its resistance to smearing and marking. This characteristic stands in contrast to previous liquid‐like biphasic thin‐film structures, which often posed challenges in integrating microchip components without the need for additional encapsulation.^[^ ^159^ ^]^

## 3 Energy Harvesters for Biomedical Applications

Energy harvesters built for biomedical purposes are a game changer in healthcare technology. Utilizing multiple energy sources within the human body to power implanted and wearable medical devices, these specialized gadgets are set to change the landscape of medical diagnostics and therapies. This subtopic investigates the tremendous influence of energy harvesting in extending the lifetime and functionality of important biomedical technology ranging from pacemakers to medication delivery systems. In this context, we will look at the underlying concepts, current breakthroughs, and future possibilities of energy harvesters designed for the healthcare industry.

### 3.1 Self‐Powered Epidermal Electronics

Various types of epidermal electronics, including electronic tattoos^[^ ^160^, ^161^, ^162^ ^]^ and electronic skin,^[^ ^92^, ^140^, ^163^ ^]^ have received enormous attention for healthcare monitoring due to their lightweight, flexibility, tissue‐like softness, skin‐conformal ability, and biocompatibility.^[^ ^164^ ^]^ Furthermore, they can detect target molecules through biofluids (e.g., sweat, blood, saliva, or ISF).^[^ ^164^, ^165^ ^]^ Energy harvesters have been developed on epidermal electronics.^[^ ^166^ ^]^ The critical challenges of the epidermal energy harvesters are the flexibility that can tolerate skin deformations without performance degradation. Here, we reported a case study that combines flexible energy harvesters and epidermal electronics in biomedical applications.

**Figure 10:**
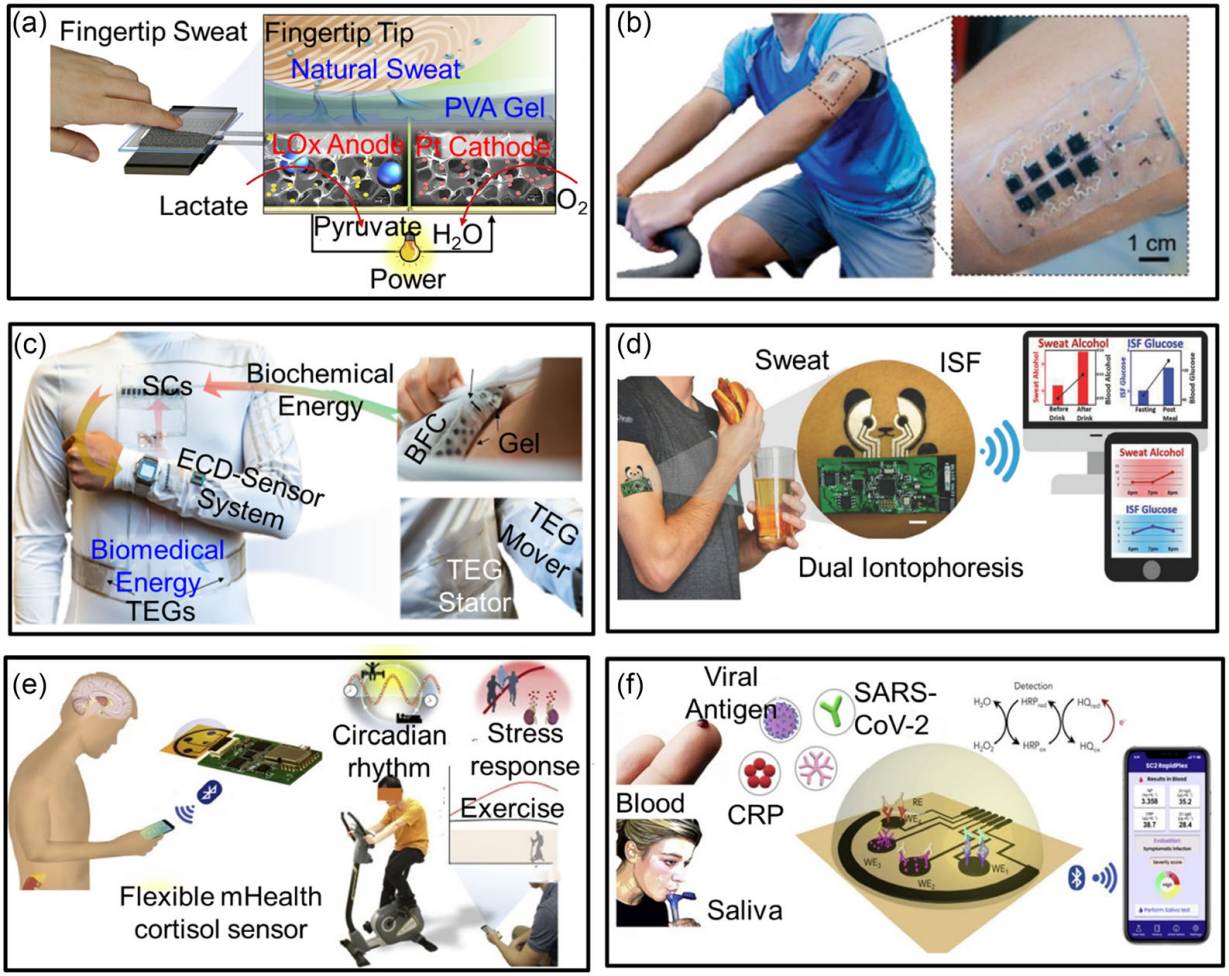
Wireless epidermal electronics for sweat analysis. a) Flexible BFCs. Reproduced with permission.^[^ ^168^ ^]^ Copyright 2021, Elsevier. b) The printed sweat‐based wearable supercapacitors were mounted on the arm. Reproduced with permission.^[^ ^170^ ^]^ Copyright 2021, Wiley‐VCH. c) System diagram of an integrated E‐textile microgrid powering a liquid‐crystal display or an electrochromic display–sensor system. Reproduced under the terms of the CC‐BY Creative Commons Attribution 4.0 International license (https://creativecommons.org/licenses/by/4.0).^[^ ^171^ ^]^ Copyright 2021, The Authors, published by Springer Nature. d) Depiction of wearable iontophoretic biosensor for glucose and alcohol sensing. Reproduced with permission.^[^ ^172^ ^]^ Copyright 2018, Wiley‐VCH. e) Integrated wireless graphene‐based sweat stress sensor for dynamic and noninvasive stress hormone analysis. The insets represent a cortisol circadian rhythm, an affinity‐based electrochemical cortisol sensor construction and sensing strategy, and a conceptual illustration of stress response monitoring by wirelessly tracking a subject's cortisol level with a cellphone. Reproduced with permission.^[^ ^173^ ^]^ Copyright 2020, Elsevier. f) Schematic illustration of the SARS‐CoV‐2 RapidPlex multisensory platform for detecting SARS‐CoV‐2 viral proteins, antibodies, and C‐reactive protein. Reproduced with permission.^[^ ^174^ ^]^ Copyright 2020, Elsevier.

BFC is a widely used bioenergy harvester in epidermal electronics as they can directly capture biological fuels (e.g., lactate, glucose, or ascorbic acid) from bodily fluids for electrocatalytic reactions.^[^ ^29^, ^167^, ^168^ ^]^ In addition, their efficient device configuration, including a carbon‐based anode, catalytic cathode, and fluidic or solid channels for biofuel supply, is feasible to operate in flexible and stretchable formats.^[^ ^40^, ^41^, ^169^ ^]^ Wang's group demonstrated an efficient touch‐based lactate BFC powered by natural fingertip sweat (**Figure** **10**a).^[^ ^168^ ^]^ Such a device can continuously generate hundreds of mJ of energy and sustainably power the sensor and electrochromic display, representing a more efficient and reliable approach than other reported harvesters (Figure 10b).^[^ ^170^ ^]^ The mechanical robustness and conformability of the system were further validated by a bending test with 20% stretching for 100 cycles. The lactate‐based BFCs could harvest ≈0.343 mW cm^−2^ and store the energy from sweat during exercise. To further increase the power output, Yin et al. reported an electronic textile microgrid system integrating BFC and TENG (Figure 10c).^[^ ^171^ ^]^ BFC and TENG can synergistically scavenge energy from sweat and human activities, generating high‐power output. BFC‐powered wearable platforms have significant potential for next‐generation self‐powered wearable bioelectronic devices.

Kim et al. developed an electronic tattoo for the simultaneous monitoring of sweat and ISF using a single wearable epidermal platform (Figure 10d).^[^ ^172^ ^]^ The epidermal e‐tattoo detected alcohol variations in sweat and glucose changes in ISF from human subjects when asked to eat or drink. A system for wireless transmission of the responses to a smartphone or laptop has been established. Gao's group fabricated a graphene‐based cortisol sensor to investigate the dynamics of cortisol in sweat (Figure 10e).^[^ ^173^ ^]^ They realized a noninvasive, real‐time, and wireless stress monitoring using an RF module (i.e., Bluetooth). It is expected to be used for mental healthcare management *via* noninvasive dynamic monitoring of cortisol in sweat. They also applied this graphene‐based wearable and wireless platform for ultrasensitive, highly selective, and ultrarapid detection of the SARS‐CoV‐2 Rapid Plex in blood and saliva (Figure 10f).^[^ ^174^ ^]^ The responses can be wirelessly transferred to a COVID‐19 telemedicine platform to assist diagnosis and evaluation of disease without the need for external batteries and other measurement instruments.

### 3.2 Smart Contact Lens

**Figure 11:**
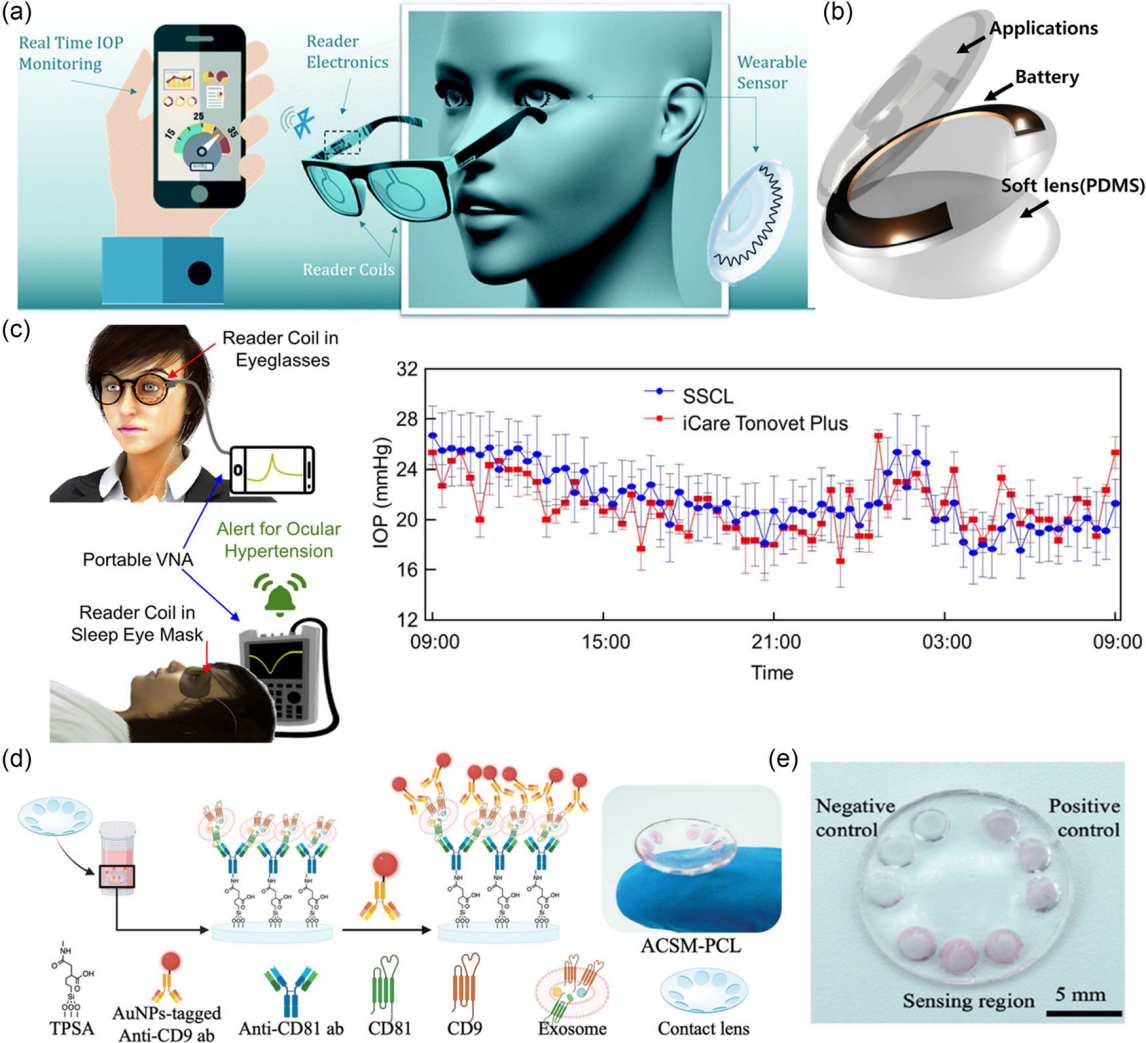
SCL for the monitoring of IOP and tear exosomes. a) Schematics of the continuous IOP monitoring system. Reproduced with permission.^[^ ^175^ ^]^ Copyright 2020, Royal Society of Chemistry. b) Schematic of battery‐embedded SCL. Reproduced with permission.^[^ ^176^ ^]^ Copyright 2018, Elsevier. c) Data acquisition scheme for the IOP monitoring during daytime and nighttime. 24 h IOP rhythm of a dog obtained from the SCL. Reproduced under the terms of the CC‐BY Creative Commons Attribution 4.0 International license (https://creativecommons.org/licenses/by/4.0).^[^ ^177^ ^]^ Copyright 2022, The Authors, published by Springer Nature. d) Schematic illustration of the colorimetric assay. The captured tear exosomes on the SCL are visualized with AuNP‐tagged anti‐CD9 antibodies. e) Photos of the ACSM‐PCL to detect tear exosomes. d,e) Reproduced with permission.^[^ ^178^ ^]^ Copyright 2022, Wiley‐VCH.

Smart contact lens (SCL) has been recently developed for precision and personalized healthcare. The contact lens has been hailed as a promising wearable platform integrated with biosensing capabilities. Compared to other biofluids (i.e., saliva, blood, sweat, or urine), the tear is considered a cleaner biofluid since it does not contain many interfering molecules that cause difficulties in improving sensitivity, selectivity, or accuracy. In these regards, many researchers focus on developing flexible SCL devices that can operate with their energy and transfer physiological information wirelessly (**Figure** **11**a).^[^ ^175^ ^]^ To achieve this goal, the SCL should include biosensors and energy harvesters on its surface (Figure 11b).^[^ ^176^ ^]^ Moreover, it should be transparent, which will not interrupt vision. For example, Lee's group developed an SCL integrated with a serpentine strain sensor for continuously monitoring intraocular pressure (IOP) in advanced glaucoma care (Figure 11c).^[^ ^177^ ^]^ Glaucoma progressively deteriorates vision without early warning signs or pain and remains the leading cause of irreversible blindness worldwide. Real‐time continuous monitoring will facilitate early‐stage diagnosis and thus provide timely treatment. To further enhance the practicability and reliability of the SCL, Lee continues to optimize their SCL prototype to achieve such IOP monitoring that can last for 24 h, even during sleep. This SCL was fabricated on a commercial lens and exhibited its intrinsic properties without alteration. During that time, Zhu et al. developed a microchamber containing SCL for the noninvasive detection of tear exosomes (Figure 11d).^[^ ^178^ ^]^ To address the power issue, they integrated a colorimetric assay for the wearable analysis of tear biomarkers in a battery‐free fashion (Figure 11e). This proof‐of‐concept work demonstrated the SCL with integrated microfluidics as a rapid, noninvasive monitoring platform for cancer prescreening or early detection.

**Figure 12:**
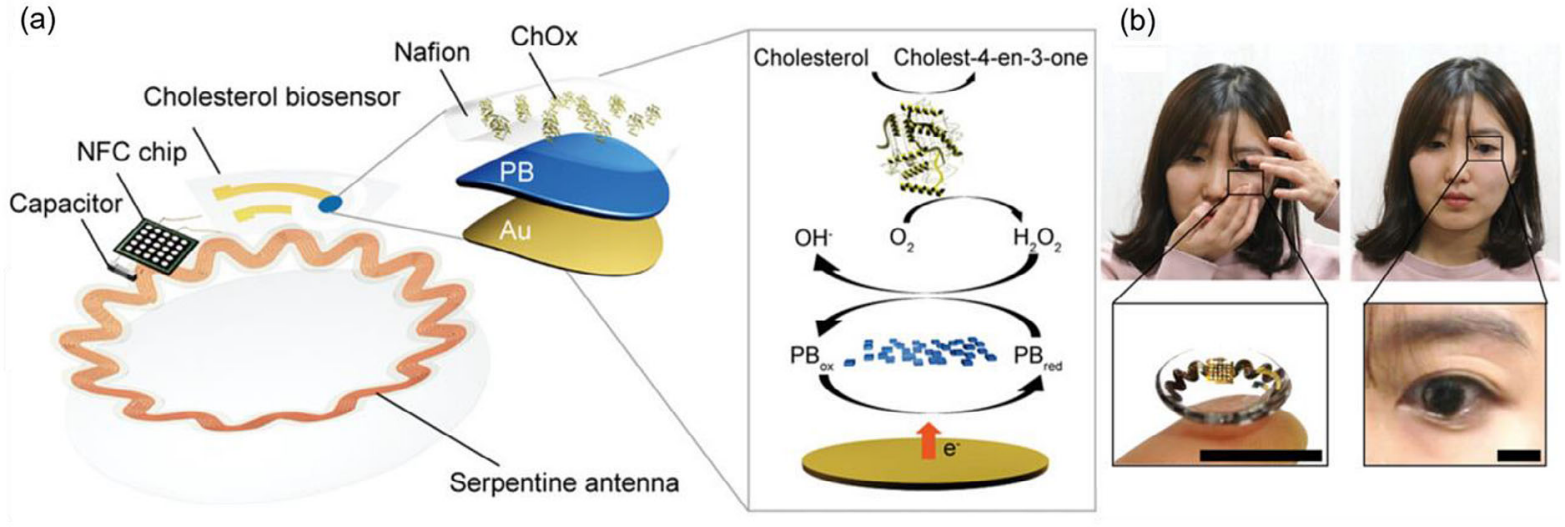
SCL for cholesterol monitoring. a) Schematic illustration of the SCL, including a cholesterol biosensor with the enzymatic reactions. b) Photographs of a female human subject wearing the lens and during the measurement using a smartphone. a,b) Reproduced with permission.^[^ ^170^ ^]^ Copyright 2022, Wiley‐VCH.

Park's group developed several SCL devices to meet different sensing capabilities, including monitoring glucose, cortisol, or cholesterol in tears.^[^ ^8^, ^170^, ^179^ ^]^ They also integrated a signal‐transferring system onto the lens for wireless communication. Specifically, they reported an SCL biosensor to track tear glucose level changes.^[^ ^8^ ^]^ Such a transparent and stretchable ocular platform comprises an LED pixel, rectifier circuit, glucose sensor, and antenna, providing wireless visualization of multiple biosignals in real time. In addition, Ku et al. reported a small graphene‐based field‐effect transistor cortisol sensor and integrated it into the contact lens.^[^ ^179^ ^]^ They developed an NFC readout system on the lens to monitor the physiological signals through a smartphone. This fully integrated SCL system allowed for wireless and battery‐free operation without any other external equipment. Based on the proof‐of‐study results with human subjects, a wireless SCL has been validated to have the potential for on‐eye applications. Song et al. used a similar strategy and developed an SCL device for noninvasive cholesterol monitoring, and such data can be wirelessly transferred to a smartphone via an NFC unit (**Figure** **12**a,b).^[^ ^170^ ^]^ Therefore, SCLs are expected to serve as noninvasive healthcare monitoring or assistive diagnostic tool for ocular diseases.

## 4 Conclusion and Perspectives

Flexible bioelectronics has advanced considerably in biomedical devices to offer the capabilities for human healthcare monitoring, early disease diagnosis, or prevention. For the last few decades, flexible bioelectronic devices have witnessed a progressive development in digital healthcare and telemedicine (**Table** **1**). Simultaneously, emerging energy harvesters (i.e., BFCs, solar cells, PENGs, and TENGs) have been developed and integrated into flexible electronic devices. They allow the devices to operate sustainably without the requirement of other external power sources. Meanwhile, RF modules have been designed and developed for electronic devices, enabling wireless communication and energy harvesting. Based on these advances, flexible bioelectric devices have been revolutionized in the biomedical field as practical medical alternatives for existing devices, offering personalized management and enhanced control over health.

**Table 1:** Development history of flexible bioelectronics field

Year	Milestone	References
2004	Development of the first flexible transistor.	[186]
2008	Introduction of flexible bioelectronic sensors.	[187]
2010	Development of flexible implantable bioelectronics.	[188]
2011	Integration of flexible bioelectronics with neural interfaces.	[189]
2015	Demonstration of flexible bioelectronic skin.	[190]
2016	Development of biocompatible materials for flexible bioelectronics.	[191]
2019	Advancements in flexible bioelectronic energy harvesting.	[192]
2020	Development of wireless communication in flexible bioelectronics.	[193]
2022	Development of bioresorbable flexible bioelectronics.	[194, 195]

Furthermore, machine learning and artificial intelligence are increasingly applied in various medical devices to improve accuracy and convenience significantly. So far, some work has already adapted machine learning to their bioelectronic devices, but much remains to be done. Intelligent wearable bioelectronics will offer multiple features, such as on‐chip computation, augmented reality, dynamic gesture recognition, and customized information presentation, while blending seamlessly with clothes and accessories. Furthermore, generating energy‐saving and self‐powered strategies will reduce or minimize the heavy need for external power sources.

Wireless interaction with the human body is also the key to the future of flexible bioelectronics electronics in both wearable and implantable applications. They can offer real‐time monitoring and timely treatments for chronic illnesses, neurological disorders, and rehabilitation. Such a platform could enable individualized and precise therapies to meet the specific needs of each patient through a self‐regulated drug delivery system by collaborating with brain stimulation and tissue engineering.

In addition to these, we may anticipate increased integration of cutting‐edge materials into flexible bioelectronics in the upcoming years, including liquid metals, perovskite materials, and organic photovoltaics. These materials have special qualities that can increase the flexibility and efficiency of energy harvesting, creating new opportunities for implantable and wearable self‐powered devices. The creation of more compact and covert wearable technology depends on the shrinking of energy harvesters. Microscale and nanoscale energy harvesters that can produce electricity from even minute physiological movements or temperature gradients are anticipated to be the subject of future study. Enzymatic energy harvesters and BFCs show great promise for powering implanted medical devices. The use of biocompatible enzymes and microorganisms to transform biological substrates (such as glucose or lactate) into electrical energy will probably be the subject of future research in this field. Using intelligent materials, such as piezoelectric polymers and shape memory alloys, can create adaptable energy harvesters that can adjust to changing environmental circumstances. Based on the wearer's activity level, ambient variables, and device needs, these systems may optimize energy generation. A major area of concentration will be on creating effective energy storage systems that work with energy harvesters. To store gathered energy for long‐term usage, research may examine improvements in energy‐dense supercapacitors or new energy storage materials. A crucial factor will be ensuring the long‐term biocompatibility and security of energy harvesting materials inside the human body. A thorough evaluation of these materials’ effects on human health over a long period of time will be done through testing and investigations.

## Conflict of Interest

The authors declare no conflict of interest.
